# Impact of Blood Collection Tubes and Sample Handling Time on Serum and Plasma Metabolome and Lipidome

**DOI:** 10.3390/metabo8040088

**Published:** 2018-12-04

**Authors:** Charmion Cruickshank-Quinn, Laura K. Zheng, Kevin Quinn, Russell Bowler, Richard Reisdorph, Nichole Reisdorph

**Affiliations:** 1 Skaggs School of Pharmacy and Pharmaceutical Sciences, University of Colorado Anschutz Medical Campus, Aurora, CO 80045, USA; Charmion.Cruickshank-Quinn@ucdenver.edu (C.C.-Q.); Kevin.Quinn@ucdenver.edu (K.Q.); Richard.Reisdorph@ucdenver.edu (R.R.); 2 Department of Medicine, St. Joseph Hospital, Denver, CO 80218, USA; Laura.Zheng@sclhealth.org; 3 Department of Medicine, National Jewish Health, Denver, CO 80206, USA; BowlerR@njhealth.org

**Keywords:** metabolomics, lipidomics, clinical, collection tubes, blood, plasma, serum

## Abstract

*Background*: Metabolomics is emerging as a valuable tool in clinical science. However, one major challenge in clinical metabolomics is the limited use of standardized guidelines for sample collection and handling. In this study, we conducted a pilot analysis of serum and plasma to determine the effects of processing time and collection tube on the metabolome. *Methods*: Blood was collected in 3 tubes: Vacutainer serum separator tube (SST, serum), EDTA (plasma) and P100 (plasma) and stored at 4 degrees for 0, 0.5, 1, 2, 4 and 24 h prior to centrifugation. Compounds were extracted using liquid-liquid extraction to obtain a hydrophilic and a hydrophobic fraction and analyzed using liquid chromatography mass spectrometry. Differences among the blood collection tubes and sample processing time were evaluated (ANOVA, Bonferroni FWER ≤ 0.05 and ANOVA, Benjamini Hochberg FDR ≤ 0.1, respectively). *Results*: Among the serum and plasma tubes 93.5% of compounds overlapped, 382 compounds were unique to serum and one compound was unique to plasma. There were 46, 50 and 86 compounds affected by processing time in SST, EDTA and P100 tubes, respectively, including many lipids. In contrast, 496 hydrophilic and 242 hydrophobic compounds differed by collection tube. Forty-five different chemical classes including alcohols, sugars, amino acids and prenol lipids were affected by the choice of blood collection tube. *Conclusion*: Our results suggest that the choice of blood collection tube has a significant effect on detected metabolites and their overall abundances. Perhaps surprisingly, variation in sample processing time has less of an effect compared to collection tube; however, a larger sample size is needed to confirm this.

## 1. Introduction

Metabolomics is part of the “omics cascade” along with genomics, transcriptomics and proteomics [1]. It reflects dynamic and rapid changes in phenotype following perturbation and arguably is most closely related to phenotype [2,3]. Metabolomics can provide valuable insight into effects of disease, genetic variation and treatment on the metabolic state of organisms [4]. 

However, one major challenge in metabolomics is the limited use of standardized blood collection, handling and processing methods when collecting samples for metabolomics analyses. While standard operating procedures are available, they are not widely adopted and many laboratories have their own quality control systems that are not universally accepted. Because mass spectrometry-based metabolomics is a highly sensitive technique, inconsistencies in sample collection and handling can create variability in the data and these variations can be readily detected. For example, human plasma is a heterogenous mixture of proteins, water, organic compounds and inorganic salts; these components can be subject to various effects during sample handling, processing and storage conditions [5,6]. 

Previous studies have evaluated the effects of collection and handling on the proteome [6,7]. To date, only a few studies have considered serum and/or plasma stability under diverse sample collection and handling conditions in the context of metabolomics. Such studies have focused on either targeted analysis, GC, or NMR-based metabolomics. For example, targeted liquid chromatography mass spectrometry (LC-MS) metabolomics was used by Yu et al. to compare the profiles of plasma and serum of 163 metabolites [8]. However, this study was limited to only a single collection followed by storage at −80 °C. Pagliaa et al. compared serum and plasma using LC-MS metabolomics but this was limited to 189 targeted compounds [9]. Another study analyzed serum and plasma at 22 °C for four time points (2, 4, 8, 24 h) using untargeted LC-MS metabolomics [10]. In that study, the authors recommended that to maintain sample stability, samples needed to be immediately placed on ice. Jobart et al. analyzed serum and plasma storage and handling conditions using two temperatures (4 °C, 22 °C) at two timepoints (1, 6 h) using NMR metabolomics [11], while Teahan et al. also used NMR metabolomics to evaluate analytical bias in serum and plasma using PCA and pattern recognition [12]. Additional studies have used NMR to evaluate storage rather than sample processing following blood collection [13]. 

Our current study expands on previous studies while focusing on sample processing rather than storage. We evaluated differences between blood collection tubes and the changes occurring within each collection tube with time at 4 °C prior to sample storage. We used untargeted LC-MS metabolomics as this approach provides vast quantities of information on small molecules and lipids due to its sensitivity and enabled us to detect a larger number of compounds compared to targeted LC-MS. Our goals were to (1) determine changes in plasma and serum compounds from blood collected in three vacutainers containing different anticoagulants and (2) to determine the effect of exposing blood to 4 °C for six time points (0, 0.5, 1, 2, 4 and 24 h) prior to centrifugation. Results can be used to guide future clinical metabolomics studies on the importance of sample collection, handling and processing prior to storage.

## 2. Results

### 2.1. Metabolite Comparisons

The vacutainer serum separator tube (SST) retained the largest number of compounds (7377) compared to the EDTA (6897) and P100 (6996) tubes. Within each tube type, minimal significant differences were observed in processing time based on ANOVA (Benjamini Hochberg FDR ≤ 0.1) across all time points (Table 1). Larger significant differences were observed due to tube type (Table 2). Based on the ‘number, regulation and tube’ comparison (ANOVA Bonferroni FWER ≤ 0.05, fold change ≥ 1.5), 410 hydrophilic and 174 hydrophobic compounds had higher abundances in the serum compared to the plasma tubes; fewer metabolites (90 hydrophilic, 57 hydrophobic) were of higher abundance in the plasma compared to the serum tubes.

### 2.2. Taxonomy Enrichment

To determine whether the significant compounds between tube types were related, taxonomy enrichment analysis was performed using the identified and annotated compounds that were significantly different (Bonferroni FWER ≤ 0.05, fold change ≥ 1.5) between pairs of tubes. This strategy captures overall differences; however, direction of regulation is not included. Nine compound classes were different (FDR ≤ 0.05) between the plasma tubes, compared to 37 between EDTA and SST and 38 between P100 and SST (Table 2). The identities of the specific classes were determined (Table 3, Supplemental Document S1); Primary and secondary alcohols accounted for most of the chemical class differences (Supplemental Document S1A). The most represented compound classes (>100 metabolites) detected in the entire dataset were secondary alcohols, glycerophospholipids, primary alcohols, carboxylic acids, saccharides, prenol lipids and cyclic alcohols (Supplemental Document S1B).

### 2.3. Clustering Based on Collection Tube and Subject

**Figure 1:**
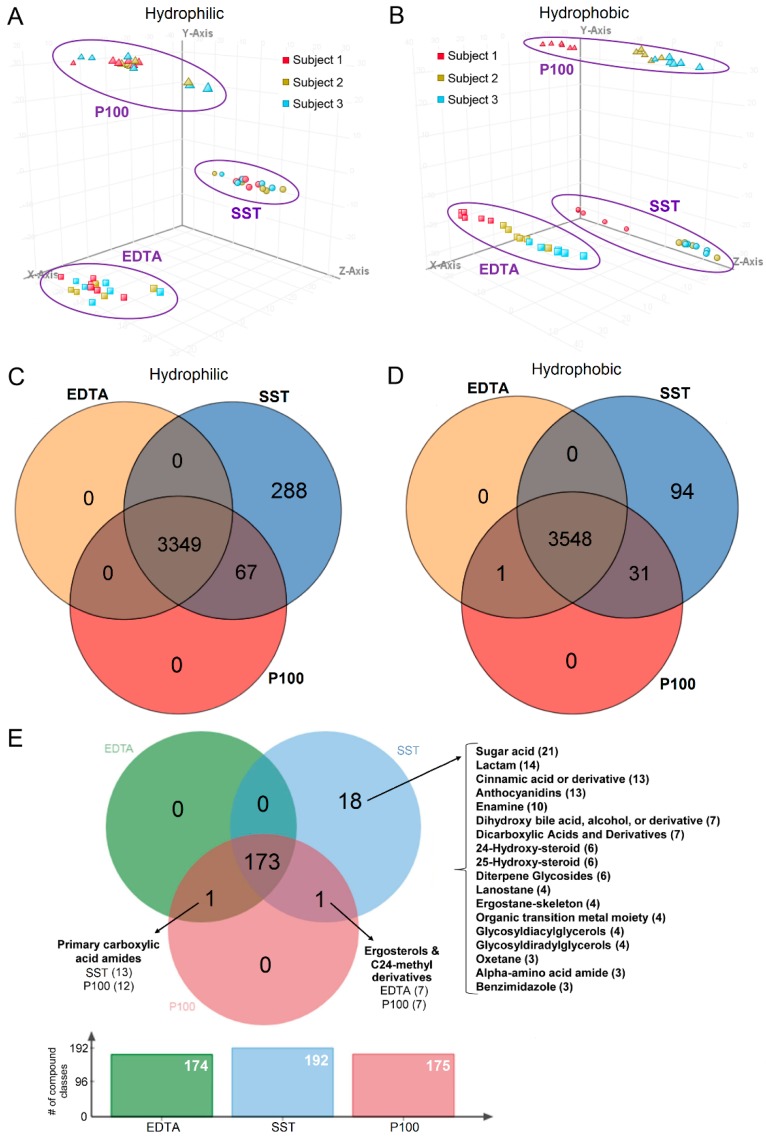
Comparison of sample tubes. (**A**) PCA plot on data shows clustering on tube type in the hydrophilic fraction. *x-axis*: PC1, 18.93%; *y-axis*: PC2, 16.83%; *z-axis*: PC3, 4.52%. (**B**) PCA plot on samples shows clustering on tube type and subject in the hydrophobic fraction. *x-axis*: PC1, 23.56%; *y-axis*: PC2, 20.9%; *z-axis*: PC3, 4.2%. PCA was performed using all metabolites prior to filtering and statistical analysis. ∆ P100, ○ SST, □ EDTA. (**C**) Overlap of metabolites in the hydrophilic fraction. (**D**) Overlap of metabolites in the hydrophobic fraction. Compounds were filtered for presence in 50% of at least one time point in each tube. (**E**) Venn diagram of taxonomy showing the overlap of compound classes that passed enrichment analysis based on FDR ≤ 0.05. The overlapping intersection shows the name of the unique compound classes, the tube type and the number of compounds from that compound class. The unique section lists the number of compound classes in SST and the number of compounds detected in that class in parenthesis.

Principal components analysis (PCA) was performed prior to filtering or statistical analysis to visually inspect clustering patterns. No time-dependent clustering was observed. In the hydrophilic fraction (Figure 1A), clustering was observed based on collection tube. In the hydrophobic fraction (Figure 1B) clustering is also observed based on collection tube; in addition, samples cluster by subjects, where subject 1 (red) clusters separately from subject 2 (brown) and subject 3 (blue).

### 2.4. Tube Overlap Based on Captured Metabolites

There was 93.5% overlap of metabolites across the serum and plasma tubes (Figure 1C,D) with more than 300 additional compounds detected in the serum tube compared to the plasma tubes. However, only 83 out of 382 compounds were matched to a database. The P100 and SST shared 98 compounds in common that were not present in the EDTA tube of which 25 compounds were matched to a name in a database. The list of these compounds is available in Supplemental Document S2. To confirm the presence of additional compounds in the serum tubes, we performed a small study comparing blank (saline) SST to blank (saline) EDTA and P100 tubes (Supplemental Document S3). There were 1247 and 1315 compounds detected in SST (gold) and SST (red/grey) tubes respectively compared to 257, 312 and 449 compounds in the EDTA (small), EDTA (large) and P100 tubes, respectively.

Taxonomy enrichment analysis was performed using all the database-annotated and identified metabolites from each of the three tube types from the blood collections to determine whether each tube captures specific compound classes (Figure 1E). There was 89.6% overlap in compound classes across all tubes. The SST contained 18 unique classes (9.3%) (Figure 1E); these included amino acid amides, dihydroxy bile acids and sugar acids (Supplemental Document S4).

### 2.5. Metabolite Abundance Differences across Tubes

**Figure 2:**
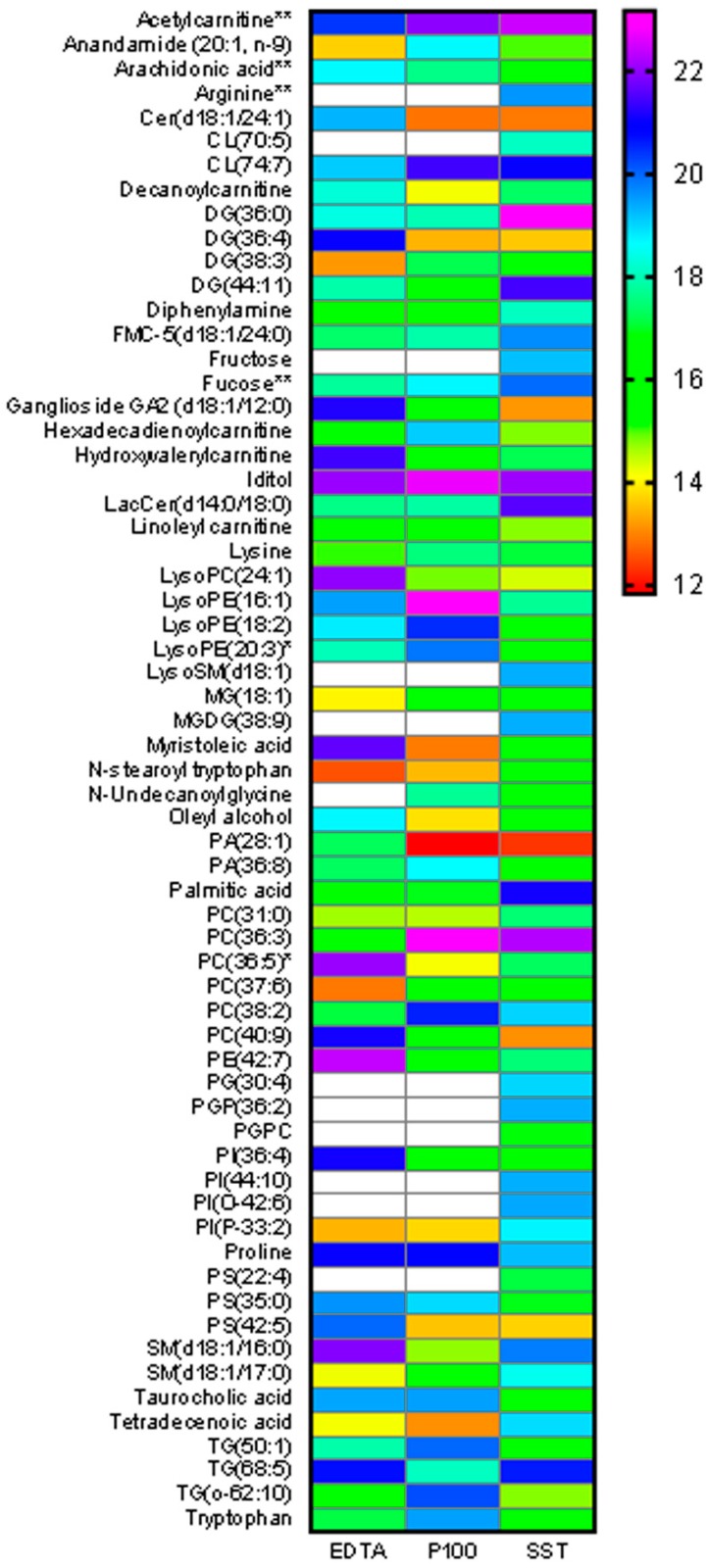
Abundance differences among blood collection tubes. The control tubes were compared to determine extraction differences using One-way ANOVA with Bonferroni FWER ≤ 0.05 and fold change ≥ 1.5. The database annotated metabolites are shown. ** indicates an identification with MSMS and standards, * indicates an identification with MSMS fragment match to NIST spectral library. *n* = 3 individuals per time point. The heatmap colors range from red (low abundance) to purple (high abundance). White indicates an abundance that was below the level of detection or filtering thresholds (missing values). Cer: ceramide, CL: cardiolipin, DG: diglyceride, PA: phosphatidic acid, PC: phosphatidylcholine, PE: phosphatidylethanolamine, PI: phosphatidylinositol, SM: sphingomyelin, TG: triglyceride.

The statistically significant compounds from the control tubes comparison were plotted to determine whether specific compounds were captured at higher abundance in a particular collection tube (Figure 2). Results show that PC(36:3) is higher in abundance in P100 and SST compared to EDTA while DG(36:0) is highest in SST. LysoPE(16:1) is highest in P100 while SM(d18:0/16:1) and LysoPC(24:1) are highest in EDTA. PC(37:6) and DG(38:3) are lowest in EDTA, oleyl alcohol is lowest in P100 and ganglioside GA2(d18:1/12:0) is lowest in SST. No general trend could be identified, suggesting that these differences are the result of random variation rather than specifically related to tube type. However, there were many compounds that were of high abundance in SST but below our limit of detection of filtering thresholds in P100 and EDTA. These are designated with white boxes in the heat map. Such compounds include arginine, fructose, LysoSM(18:1) and MGDG(38:9). Heat maps showing additional data for the amino acids, LysoPCs, sugars and carnitines are available in Supplemental Figure S1.

### 2.6. Time Trends and Changes Indicative of Metabolite Degradation, Oxidation, or Hydrolysis

**Figure 3:**
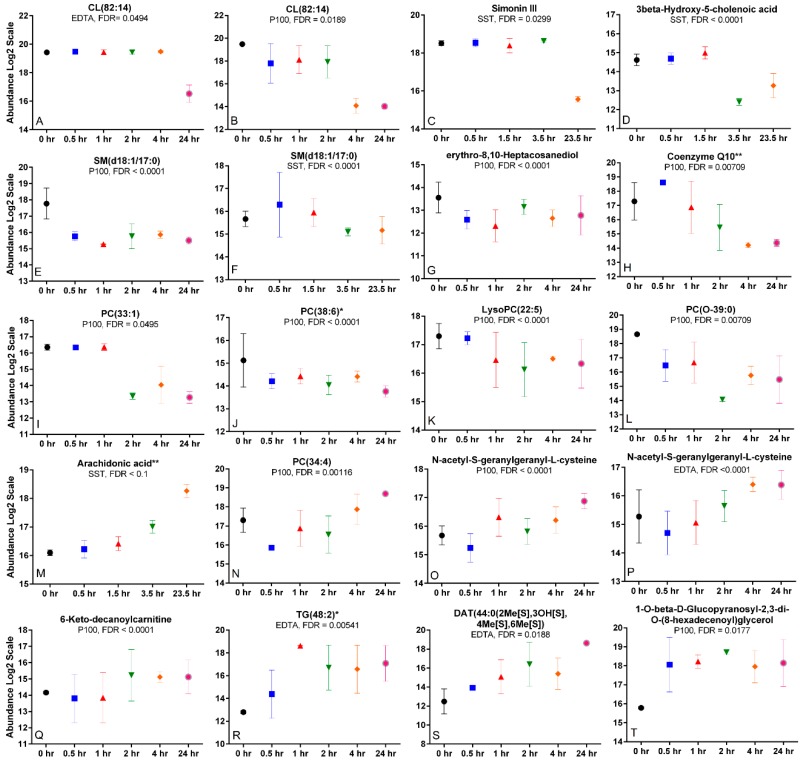
Metabolites affected by time. (**A**–**L**) Compounds whose abundance decreased and (**M**–**T**) compounds whose abundance increased with time in the indicated blood collection tube. Processing time differences are based on Repeated Measures ANOVA with FDR ≤ 0.1. * indicates identified by MSMS fragments to the NIST spectral library. ** indicates identified using standards and MSMS fragmentation. Error bars represent standard error of the mean, *n* = 3 subjects per time point.

The compounds affected by processing time with database or library matches were plotted (Figure 3A–T). Distinct time trends were observed due to processing time for the blood tubes. CL(82:14) was stable up to 4 h, then decreased >5 fold at 24 h in EDTA (Figure 3A), compared to P100 where there was a ~2-fold decrease at 30 min and then an ~8 fold decrease at 4 h (Figure 3B). A similar trend is observed in SST for simonin III (Figure 3C) with a ~4 fold decrease at 24 h and a ~6-fold decrease at 3.5 h for 3beta-hydroxy-5-cholenoic acid (Figure 3D). PC(33:1) was stable, followed by a ~6 fold decrease at 2 h and remained stable up to 24 h. Other compounds showed a gradual decrease in abundance with time such as coenzyme Q10 (Figure 3H), PC(38:6) (Figure 3J), LysoPC(22:5) (Figure 3K) and PC(O-39:0) (Figure 3L) in P100. The lipid, SM(d18:1/17:0) decreased in both P100 (Figure 3E) and SST (Figure 3F). On the contrary, other metabolites increased gradually with time, such as arachidonic acid (AA) (Figure 3M) that increased 2-fold from 0–3.5 h and then > 4-fold up to 23.5 h in the serum tube and TG(48:2) (Figure 3R) that increased in the EDTA. 

### 2.7. Differing Time Trends across Tubes

Supplemental Figure S2 shows the overlay of twelve compounds and how they behave across the three collection tubes. CL(82:14) is relatively stable over time in SST, decreases at 30 min and again at 4 h in P100, while in EDTA, it decreases at 4 h. Arachidonic acid is stable in EDTA, slowly increases with time in SST and increases at 2 h in P100. Conversely, betaine, cortisol, theobromine, LysoPC(18:1), omega-hydroxydodecanoic acid and palmitoylcarnitine were stable for 24 h in all three blood collection tubes.

## 3. Discussion

Clinical studies may use either plasma or serum for their analyses; however, these biological fluids are inherently different. We observed that SST, which is used to prepare serum, retained a higher number and more unique compounds compared to EDTA and P100 tubes used to collect plasma. In fact, this effect has been demonstrated in a targeted metabolomics study [8], as well as in proteomics [14] and genomics [15]. It could be argued that the clot activator and gel contained in the SST is contributing to the additional compounds found in serum, producing more “unique” compounds, as observed with the 382 unique compounds observed, with only 83 matching to a compound name in a database. Also, some of the additional compounds may be protein breakdown products since serum (compared to plasma) has a higher concentration of thromboglobulins and activation peptides released during platelet activation [16]. In our own studies (unpublished) we have observed differences in metabolome profiles based on the type of serum tube used for sample collection.

The choice of blood collection tube will largely depend on the technology being used. In proteomics analysis, peptides in serum are dramatically different from those in plasma [6]. In DNA analysis, EDTA plasma is the typical choice as it inhibits DNAse activity, while heparin is unstable and inhibits PCR [17,18]. Heparin collection tubes are commonly used in NMR studies because EDTA causes interference in the NMR spectrum and masks many of the metabolite peaks [19]. Comparatively, EDTA plasma is typically used in LC-MS studies since LC-MS is more sensitive to even small biological changes. For example, SSTs are generally left on the bench for an unspecified amount of time, which can result in metabolite abundance differences, as observed in our study.

Overall, we observed that the metabolites detected were more sensitive to tube differences compared to processing time. This may be due to a limitation of our small sample size. However, we did observe that arachidonic acid (AA) increased in abundance with time in SST. Arachidonic acid is formed in one of two ways; (1) the enzyme phospholipase A2 cleaves AA from membrane phospholipids or (2) in a two-step process when phospholipase A2 frees a diglyceride from a phospholipid that is then hydrolyzed by the enzyme diacylglycerol lipase to form AA [20]. This gradual increase in abundance with time also suggests release of AA from the platelets during blood clot formation.

Of the metabolites affected by time, there were compounds whose abundance decreased significantly within the first 30 min of being placed on ice and in a 4 °C refrigerator prior to processing. At least in the case of these metabolites, it would be crucial to process plasma and serum rapidly upon collection to ensure maximum detection and reproducibility via mass spectrometry. There were also serum and plasma metabolites whose abundance remained relatively stable for the first four hours and subsequently decreased between time points 4 h and 24 h. This finding is supported by Jobard et al., who showed that metabolites in serum and plasma were of acceptable quality after the collected samples were kept at 4 °C for 6 h [11]. Two examples in our study, include CL(82:14), a double phospholipid constituting 20% of the total lipid in the inner mitochondrial membrane and is important in blood clotting and simonin III, an oligosaccharide present in potato and sweet potato and has been suggested as a marker of potato consumption. These compounds are relevant in disease marker studies and nutritional studies. Of note, Jobard et al. [11] observed a statistically significant decrease in glucose with time in both serum and plasma. We too observed a non-significant yet decreasing trend for glucose in EDTA plasma and a statistically significant decrease in glucose (FDR = 0.0363) with time in serum. This is also supported by other studies [21,22] demonstrating the depletion of glucose with time, particularly at 24 h, in serum and plasma.

Based on our abundance comparisons in the control tubes, specific compounds were elevated in plasma compared to serum and vice versa. For example, the LysoPEs and LysoPC(24:1) were more abundant in EDTA and P100 compared to SST. A study by Yu et al. [8] observed increases in LysoPCs including LysoPC(16:0) and LysoPC(17:0) in serum compared to plasma. We too observed increases in these compounds, however they did not reach statistical significance. Unlike the authors who also observed increases in LysoPC(18:0) and LysoPC(18:1) in serum, we observed similar abundances in both lipids in our serum and plasma tubes.

In addition to lipids, we evaluated amino acids and fatty acids and compared our results to other studies. Liu et al. performed GC-MS on serum and plasma and reported that arachidonic acid (AA), glucose and amino acids were most abundant in serum compared to plasma [23]. While we observed an increase in AA with time in the serum tube, unlike the authors, the abundance of AA was higher in our plasma tubes compared to the serum tubes. In agreement with their study, arginine was also more abundant in our serum compared to plasma. This is similar to Yu et al. [8] who also observed an increase in arginine in serum compared to plasma. However, unlike these two studies, we observed an abundance in proline, creatine and tryptophan in plasma compared to serum samples and detected comparable levels of leucine and valine across tubes. These slight differences may be attributed to differences in platform (NMR vs. GC-MS vs. LC-MS), ionization mode since some compounds ionize better in negative rather than in positive mode, or sample handling since the methods used to extract the compounds were different across studies. This therefore highlights the importance of using standardized protocols to collect and handle clinical metabolomics samples.

In summary, our findings support the recommendation for prompt processing of blood samples within 30 min of collection. However, for some metabolites in serum, leaving the samples at 4 °C for up to 4 h prior to processing still produces metabolites of accurate and acceptable quality. Therefore, prior knowledge on the types of compounds expected to be observed in your metabolomics study is important, as this will help determine collection tube, optimal processing time and metabolomics platform (LC-MS vs. GC-MS vs. NMR).

This pilot study had some limitations, including the number of subjects. However, repeated measures ANOVA enabled statistically significant differences to be explored using each subject as their own control. This however assumes a linear relationship with time for each compound that may not necessarily be true for all compounds. We were also stringent with our statistics to avoid reporting false positives but acknowledge that we may have also missed other changing compounds. Second, since the blood tubes were drawn in the order of time on the bench, the metabolome may be influenced by stress during blood collection of the first tube. Third, the effects of biological variations, such as food intake, physical activity, gender and sleep pattern affect the types of metabolites found in plasma [24,25,26,27,28,29] and were not controlled for in this study. However, the use of each subject as its own control corrected this limitation. Lastly, identification of every detected metabolite is a challenge in metabolomics due to lack of processing software that could identify metabolites with unknown chemical nature or those with low signal intensities [30,31]. Despite limitations, our study supports the importance of choosing the most appropriate blood collection tube, processing and handling time during the design and implementation of clinical metabolomics studies.

## 4. Materials and Methods

### 4.1. Ethics Statement

All methods were performed in accordance with the relevant guidelines and regulations of National Jewish Health. The current study was focused on optimizing collection and preparation methods as part of internal quality improvement (i.e., method development) and was therefore considered exempt by the National Jewish Human Subjects Research review process. No identifiable information was provided by the subjects and no clinical or personal information was used.

### 4.2. Study Population and Sample Collection

Non-fasted blood from 3 healthy volunteers (40–50 years old, 2 males, 1 female) was collected in 3 types of blood collection tubes: BD Vacutainer serum separator tube^®^ (SST) (Franklin Lakes, NJ, USA) (10 mL, 16 × 125 mm) containing micronized silica particles (clot activators) and a double gel separator to obtain serum (BD product # 367985); ethylenediaminetetraacetic acid (EDTA) vacutainer (Franklin Lakes, NJ, USA) (10 mL, 16 × 100 mm) containing spray-dried K_2_ EDTA anticoagulant to obtain plasma (BD product # 366643); BD P100 vacutainer (Franklin Lakes, NJ, USA) (8.5 mL, 16 × 100 mm) containing spray-dried K_2_ EDTA anticoagulant, proprietary proteinase inhibitors and a mechanical separator to obtain plasma (product # 366448).

The blood tubes were drawn in the order of time on the bench. Specifically, the first tube drawn was time 0, the second tube 30 min and so forth. Per manufacturer recommendations, the SSTs required 30 min at room temperature prior to centrifugation to allow clot formation. Blood samples in the plasma tubes were put on ice during collection and placed in the refrigerator at 4 °C for incubation for 0, 0.5, 1, 2, 4, or 24 h. Blood samples in the serum tubes were put on ice during collection and placed in the refrigerator at 4 °C for incubation for 0, 0.5, 1.5, 3.5, or 23.5 h. Blood tubes were inverted several times after sample draw and centrifuged at 1300× *g* at 4 °C for 15 min. The resulting serum or plasma was aliquoted into pre-chilled microcentrifuge tubes and stored at −80 °C prior to metabolomics sample preparation and analysis. 

### 4.3. Reagents and Standards

Solvents used for extraction of metabolites and mass spectrometry analysis were of LC/MS-grade as follows: water and isopropyl alcohol from Honeywell Burdick & Jackson (Muskegon, MI, USA); methyl tert-butyl ether from J.T. Baker (Central City, PA, USA); acetonitrile, methanol and formic acid from Fisher Scientific (Fair Lawn, NJ, USA); standards from Avanti Polar Lipids Inc. (Alabaster, AL, USA) and Sigma Aldrich (St. Louis, MO, USA); glass pipette tips, plastic pipette tips and microcentrifuge tubes from Fisher Scientific (Fair Lawn, NJ, USA); Pyrex glass culture tubes from Corning Incorporated (Corning, NY, USA).

### 4.4. Sample Preparation

For quality control (QC) purposes, single aliquots of plasma and serum from all of the biological samples were pooled and re-aliquoted per untargeted clinical metabolomics guidelines [32]. These were used as sample preparation and instrument QCs and were prepared and analyzed alongside experimental samples. Sample preparation was randomized and performed as previously described with some modifications [33,34]. Plasma, serum and QC samples were thawed at room temperature and briefly vortexed. 100 μL of each sample were transferred to a 1.5 mL microcentrifuge tube and kept at 0 °C. 10 μL of hydrophobic and hydrophilic standards and spikes at room temperature were added and the samples vortexed for 10 s. 400 μL of ice-cold methanol was added to precipitate proteins. Tubes were vortexed for 10 s and then centrifuged for 15 min at 0 °C at 18,000× *g*. The supernatant was transferred to a clean glass culture tube using a plastic pipette. 

Samples were dried in glass culture tubes placed under N_2_ at 35 °C for approximately 1 h. Using a glass pipette, 3 mL of methyl tert-butyl ether (MTBE) was added to the dried methanol residue in each glass culture tube. Tubes were vortexed for 30 s. 750 μL of water was added and tubes were vortexed for 10 s. Tubes were centrifuged for 10 min at room temperature at ~200× *g*. 2.5 mL of the resulting MTBE layer (hydrophobic fraction) was transferred to a new glass culture tube; the remaining layer was the hydrophilic fraction. 3.0 mL of MTBE was added to the remaining hydrophilic fraction and tubes were vortexed for 10 s. These tubes were again centrifuged for 10 min at room temperature at ~200× *g*. 3.0 mL of MTBE was aspirated and combined with the first MTBE layer. The MTBE fractions were dried under N_2_ at 35 °C and immediately re-suspended in 200 μL of methanol. Each tube was vortexed for 5 s, transferred to a glass auto-sampler vial with a glass insert using a Pasteur pipette. Samples were stored at −80 °C.

The hydrophilic fractions were dried under N_2_ at 35 °C. 100 μL of water and 400 μL of ice-cold methanol were added to the dried hydrophilic fraction. Tubes were vortexed for 10 s and spun immediately at ~200× *g* for 1 min. Supernatants from each tube were transferred to a 1.5 mL microcentrifuge tube using a Pasteur pipette. These tubes were then stored at −80 °C for 25 min then spun for 15 min at 0 °C and 18,000× *g*. The supernatant was transferred to a new 1.5 mL microcentrifuge tube using a plastic pipette. These tubes were dried in a vacuum centrifugal concentrator at 45 °C and re-suspended in 100 μL of 95:5 water: acetonitrile. Each tube was vortexed for 30 s and transferred to a glass auto-sampler vial with a glass insert using a plastic pipette tip. Samples were stored at −80 °C.

### 4.5. Liquid Chromatography

All samples were randomized prior to instrument analysis for both the hydrophobic and hydrophilic fraction. The hydrophobic fraction was analyzed using an Agilent Zorbax Rapid Resolution HD SB-C18, 1.8 micron, 2.1 × 100 mm analytical column on an Agilent 1290 series pump. Injection volume was 4 µL. HPLC flow rate was 0.7 mL/min with the following mobile phases: mobile phase A was water with 0.1% formic acid and mobile phase B was 60:36:4 isopropyl alcohol:acetonitrile:water with 0.1% formic acid. The gradient was as follows for positive mode: 0.0–1.0 min 30–70% B, 1.0–7.92 min 70–100% B, 7.92–10.4 min 100% B, 10.4–10.5 min 100–30% B, 10.5–15.1 min 30% B. Autosampler tray temperature was set to 4 °C and column temperature was set to 60 °C. 

The hydrophilic fraction was analyzed on an Agilent 1200 series pump using a Phenomenex Luna NH_2_ HILIC, 5 µm, 2 × 250 mm analytical column. 2 µL was injected with a flow rate of 0.5 mL/min. Mobile phase A was 50% ACN with 20 mM ammonium acetate pH 9.45 and mobile phase B was 100% ACN. Gradient elution was as follows: 0–4 min 90% B, 4.0–15.0 min 90–28% B, 15.0–15.01 min 28–0% B, 15.01–20.0 min 0% B, 20.0–20.01 min 0–90% B, 20.01–30.0 min 90% B. Autosampler tray temperature was set to 4 °C and column temperature was set to 20 °C.

### 4.6. Mass Spectrometry (MS)

The hydrophobic fraction MS conditions were as follows: Agilent 6220 Time-of-Flight (TOF)-MS with dual ESI source, scan rate 2.02 spectra/s, mass range 60–1600 m/z, gas temperature 300 °C, gas flow 12.0 L/min, nebulizer 30 psi, skimmer 60 V, capillary voltage 4000 V, fragmentor 120 V, reference masses 121.050873 and 922.009798 (Agilent reference mix).

The hydrophilic fraction MS conditions were as follows: Agilent 6520 Quadrupole Time-of-Flight (Q-TOF)-MS in positive ionization mode with ESI source, mass range 50–1700 m/z, scan rate 1.41 spectra/s, gas temperature 300 °C, gas flow 10.0 L/min, nebulizer 25 psi, skimmer 65 V, capillary voltage 4000 V, fragmentor 125 V, reference masses 121.050873 and 922.009798 (Agilent reference mix, Santa Clara, CA, USA).

### 4.7. Tandem Mass Spectrometry

The chromatographic method was replicated for LC-MS/MS analysis using 10, 20 and 40 eV collision energies on an Agilent 6520 Q-TOF with a scan rate 3.01 spectra/s, 2.591 s cycle time, 4 m/z isolation width and 1 min delta retention time. Fragmentation data was exported to the freely available NIST MS Search v.2.2g GUI program [35] (NIST, Gaithersburg, MD, USA). Fragments were matched to reference standards from the NIST14 and NIST17 MSMS spectral libraries [36]. Identifications are Metabolomics Standards Initiative (MSI) level 1 or 2 (where indicated with * or ** respectively) based on the proposed minimum reporting by Sumner et al. [37].

### 4.8. Data Processing of QCs and Samples

Prepared QC samples were injected after every five samples. The spectral data were evaluated for reproducibility as follows. Retention time and peak area CVs for spiked internal standards and endogenous compounds were examined to ensure <10% coefficient of variation for QC samples (Supplemental Document S5). Endogenous compounds were randomly selected based on their presence in all samples and their distribution throughout the retention time range of the chromatogram. Signal intensity of total ion chromatograms were examined to ensure reproducibility across the retention time range; variation across the largest range was < 10% corresponding to < 0.3 min retention time shift. HPLC pressure curves were < 1% CV. QCs were plotted with the samples in a PCA to visually inspect their clustering and to ensure that their variability was less than that observed in the samples (Supplemental Document S5). This is based on recent recommendations by Broadhurst et al. who suggest using a pooled QC created from all the biological test samples [32] and monitoring their variations in the datasets.

Features (ions, adducts, clusters) were collapsed into compounds as described below. The duplicate injections (A, B) of the serum and plasma samples underwent data extraction in MassHunter Profinder software (Agilent, version B.08.00). A recursive workflow comprising the ‘Find by Molecular Feature (MFE)’ and the ‘Find by Ion (FbI)’ algorithms was applied. The ‘MFE’ parameters were as follows: 1–2 charge state with +H, +Na, +NH_4_ and +K adducts and dimers in positive mode; absolute height >3000 counts. Duplicate injections were used to reduce missing values in combination with the ‘FbI’ algorithm to remine the data. This algorithm performs a targeted extraction by mining the ions at their respective retention times based on the MFE extraction results from the first step using reproducible ‘molecular features’ across sample files. The ‘FbI’ parameters were: 1–2 charge state with +H, +Na, +NH_4_ and +K adducts and dimers in positive mode; absolute height >3000 counts. Spectral data were imported into Mass Profiler Professional software version 14.9 (MPP, Agilent) for filtering, statistical analysis and metabolite annotation. The ‘A’ injections were subsequently used for further analysis by importing the spreadsheet into InfernoRDN [38] for imputation using singular value decomposition (SVD) [38,39,40] to reduce missing values that may arise from peak extraction errors. The imputed data was imported into MPP as ‘generic.’ Compounds were quality control filtered for presence in 50% of at least one time point or 50% of at least one blood tube type.

### 4.9. Metabolite Annotation

ID Browser within the MPP software was used to annotate metabolites in two passes. The first pass matched compounds to an in-house mass, retention time and MSMS library comprising > 700 authentic standards. The second pass was used to annotate compounds that were not present in the in-house library. This involved annotating with an in-house database comprising HMDB, Lipid Maps and KEGG with ppm error ≤ 10 and database score ≥ 45. The database annotations were limited to the best 10 matches. Spectra were manually investigated to improve confidence in the annotations. Compounds with MSMS spectra were matched to the NIST14 MSMS and NIST17 MSMS spectral libraries [35,36] as described in the tandem mass spectrometry section in methods.

### 4.10. Statistical Analysis 

Statistical analysis was performed using Mass Profiler Professional version 14.9 (MPP, Agilent). A repeated measures ANOVA with multiple testing correction using Bonferroni Family-wise error rate (FWER) ≤ 0.05 was performed across time points for each tube type (*n* = 3 subjects/tube) to determine time point differences across each blood collection tube. A one-way ANOVA was performed across the blood collection control tubes (0 h) using Benjamini Hochberg FDR ≤ 0.1 to determine differences across tubes. Statistically significant metabolites were filtered using fold change cutoffs as indicated. Venn diagrams and principal components analysis (PCA) was performed in MPP. Heat maps and time series plots were plotted using GraphPad Prism 7.05. Enrichment analysis for chemical classification was performed using HMDB and Lipid Maps taxonomy within MBRole 2.0 [41]. Only chemical categories with ≥ 3 hits and that passed FDR correction ≤0.05 are reported.

The mass spectrometry data from this publication is available at Metabolomics Workbench database http://www.metabolomicsworkbench.org/ (Study ID: ST001099, Study doi: 10.21228/M8P40G).

## 5. Conclusions

Overall, we found that some metabolites degrade as early as 30 min at 4 °C following blood draw, others are stable for up to four hours, while some remain unaffected. Lipids, particularly sphingomyelins and LysoPCs are affected by processing time. Even within chemical classes, some compounds are affected while other functionally or structurally similar ones are not. To minimize variation, we suggest processing plasma and serum within 30 min of sample collection and meticulously following published protocols for clinical metabolomics.

## Figures and Tables

**Table 1:** Metabolites that changed with time in the collection tubes. Samples (*n* = 3 subjects per time point for each tube) were collected on ice and placed in the refrigerator at 4 °C to incubate. MS analysis was performed in positive ionization mode. * Number of compounds post-filtering (present in 50% of at least one tube type). ^†^ Differences among time points were identified using repeated measures ANOVA across all time points, adjusted for multiple testing using Benjamini Hochberg FDR ≤ 0.1.

Blood Processing Time Differences						
	SST (Serum)		P100 (Plasma)		EDTA (Plasma)	
	* Number of Compounds	^†^ Significant in SST	* Number of Compounds	^†^ Significant in P100	* Number of Compounds	^†^ Significant in EDTA
Hydrophilic	3704	30	3416	44	3349	43
Hydrophobic	3673	16	3580	42	3548	7
**Total**	**7377**	**46**	**6996**	**86**	**6897**	**50**

**Table 2:** Comparison of metabolites extracted from various collection tubes at the control time point. Metabolites in the control plasma tubes (P100, EDTA, 0 min post-collection) and control serum tubes (SST, 30 min post-collection) were compared to identify differences in metabolite abundance across tubes. * Chemical classes with FDR ≤ 0.05 and ≥ 3 matched entities following taxonomy enrichment. ^†^ ANOVA with Tukey HSD post hoc test, Bonferroni FWER ≤ 0.05 and fold change ≥ 1.5.

Blood Collection Tube Differences					
Comparison	Fraction	^†^ Significant Compounds	^†^ Total	Number, Regulation and Tube	Number of Classes Affected *
P100 vs. EDTA	Hydrophilic	177	305	134 ↑ in P100; 43 ↑ in EDTA	9
	Hydrophobic	128		76 ↑ in P100; 52 ↑ in EDTA	
EDTA vs. SST	Hydrophilic	488	719	410 ↑ in SST; 78 ↑ in EDTA	37
	Hydrophobic	231		174 ↑ in SST; 57 ↑ in EDTA	
P100 vs. SST	Hydrophilic	433	622	343 ↑ in SST; 90 ↑ in P100	38
	Hydrophobic	189		145 ↑ in SST; 44 ↑ in P100	

**Table 3:** Taxonomy enrichment results. Repeated measures ANOVA with Bonferroni FWER ≤ 0.05 and fold change ≥ 1.5 was used to identify differences between the P100, EDTA and SST. The EDTA and P100 control plasma tubes were at 0 h post-collection. The SST serum control tube was 30 min post-collection to allow clot formation. The significant metabolites from each comparison were exported to MBRole to determine whether certain chemical classes were more predominantly captured by specific tubes. The top 15 significant chemical taxonomy classes for each comparison, where applicable, is listed numerically (# in set) based on total number of compounds in each class and FDR ≤ 0.05. The complete list is available in Supplemental Document S1 (Supplementary Materials).

Comparison	Chemical Taxonomy	Category	# in Set	*p*-Value	FDR
P100 vs. EDTA	Primary alcohol	HMDB	17	2.63 × 10^−4^	1.21 × 10^−2^
P100 vs. EDTA	Secondary carboxylic acid amide	HMDB	11	2.04 × 10^−4^	1.21 × 10^−2^
P100 vs. EDTA	Carboxamide group	HMDB	11	7.48 × 10^−4^	2.29 × 10^−2^
P100 vs. EDTA	Allyl alcohol	HMDB	7	5.93 × 10^−4^	2.18 × 10^−2^
P100 vs. EDTA	Amino Acids, Peptides and Analogues	HMDB	7	1.99 × 10^−3^	4.58 × 10^−2^
P100 vs. EDTA	Fatty Alcohols	HMDB	5	1.21 × 10^−4^	1.11 × 10^−2^
P100 vs. EDTA	N-acyl-amine	HMDB	5	1.35 × 10^−3^	3.55 × 10^−2^
P100 vs. EDTA	Lysophosphatidylethanolamines	HMDB	4	1.53 × 10^−6^	2.82 × 10^−4^
P100 vs. EDTA	Sphingomyelins	Lipid Maps	3	4.31 × 10^−5^	2.11 × 10^−3^
EDTA vs. SST	Secondary alcohol	HMDB	44	1.68 × 10^−4^	4.75 × 10^−3^
EDTA vs. SST	Primary alcohol	HMDB	32	3.61 × 10^−8^	1.09 × 10^−5^
EDTA vs. SST	Glycerophospholipids	Lipid Maps	24	4.36 × 10^−4^	3.23 × 10^−2^
EDTA vs. SST	1,2-Diol	HMDB	21	3.54 × 10^−3^	2.97 × 10^−2^
EDTA vs. SST	Cyclohexane	HMDB	18	1.04 × 10^−3^	1.31 × 10^−2^
EDTA vs. SST	Secondary carboxylic acid amide	HMDB	17	1.42 × 10^−5^	7.15 × 10^−4^
EDTA vs. SST	Carboxamide group	HMDB	17	9.65 × 10^−5^	3.24 × 10^−3^
EDTA vs. SST	Prenol Lipids	HMDB	17	1.61 × 10^−3^	1.58 × 10^−2^
EDTA vs. SST	Saccharide	HMDB	16	3.11 × 10^−3^	2.68 × 10^−2^
EDTA vs. SST	Bicyclohexane	HMDB	11	1.31 × 10^−3^	1.41 × 10^−2^
EDTA vs. SST	Allyl alcohol	HMDB	10	1.73 × 10^−4^	4.75 × 10^−3^
EDTA vs. SST	Sesterterpene	HMDB	10	1.99 × 10^−4^	5.01 × 10^−3^
EDTA vs. SST	Decaline	HMDB	10	4.44 × 10^−3^	3.12 × 10^−2^
EDTA vs. SST	Choline	HMDB	9	2.13 × 10^−3^	1.95 × 10^−2^
EDTA vs. SST	Quaternary ammonium salt	HMDB	9	4.11 × 10^−3^	3.12 × 10^−2^
P100 vs. SST	Secondary alcohol	HMDB	37	1.51 × 10^−3^	1.84 × 10^−2^
P100 vs. SST	Primary alcohol	HMDB	27	1.04 × 10^−6^	3.17 × 10^−4^
P100 vs. SST	Glycerophospholipids	Lipid Maps	20	1.56 × 10^−3^	2.03 × 10^−2^
P100 vs. SST	1,2-Diol	HMDB	19	3.84 × 10^−3^	3.45 × 10^−2^
P100 vs. SST	Prenol Lipids	HMDB	17	3.49 × 10^−4^	8.42 × 10^−3^
P100 vs. SST	Cyclohexane	HMDB	17	6.05 × 10^−4^	1.04 × 10^−2^
P100 vs. SST	Cyclic alcohol	HMDB	15	2.91 × 10^−4^	8.42 × 10^−3^
P100 vs. SST	Secondary carboxylic acid amide	HMDB	14	1.64 × 10^−4^	7.15 × 10^−3^
P100 vs. SST	Carboxamide group	HMDB	14	7.69 × 10^−4^	1.12 × 10^−2^
P100 vs. SST	Saccharide	HMDB	14	5.72 × 10^−3^	4.09 × 10^−2^
P100 vs. SST	Bicyclohexane	HMDB	11	4.36 × 10^−4^	9.50 × 10^−3^
P100 vs. SST	Sesterterpene	HMDB	10	6.68 × 10^−5^	5.67 × 10^−3^
P100 vs. SST	Decaline	HMDB	10	1.71 × 10^−3^	2.01 × 10^−2^
P100 vs. SST	Drimane-skeleton	HMDB	8	6.15 × 10^−4^	1.04 × 10^−2^
P100 vs. SST	Allyl alcohol	HMDB	8	1.41 × 10^−3^	1.79 × 10^−2^

## Supplementary Materials

The following are available online at http://www.mdpi.com/2218-1989/8/4/88/s1, Document S1: (A) Taxonomy enrichment results for the statistically significant compounds across tubes. (B) Taxonomy enrichment results for all compounds across all tubes. Document S2: Present in P100 and SST and absent in EDTA. Document S3: Compounds detected in blood collection tubes. Document S4: Unique to SST. Document S5: Quality control analysis. (A) CVs for spiked internal standards and randomly selected endogenous compounds. (B) Variation of QCs compared to samples. PCA showing clustering of the QCs compared to the biological samples. Figure S1: Abundance Trends. (A) Abundance of arachidonic acid, amino acids and sugars. Arachidonic acid, arginine, lysine, proline, tryptophan, fructose, fucose and idiol were statistically significant (FDR < 0.05). (B) Abundance of LysoPCs. LysoPC(24:1) was statistically significant (FDR < 0.05). (C) Abundance of carnitines. Acetylcarnitine, 9,12-hexadecadienoylcarnitine, 6-keto-decanoylcarnitine and linoleyl carnitine were statistically significant (FDR < 0.05). ** or * indicates identified by MSMS and standards or MSMS to the NIST spectral library, respectively. Figure S2: Compound Overlay. Time trends of twelve compounds overlaid in three blood collection tubes. ** indicates MS1 confirmed mass, retention time and MSMS by matching to purchased standards. * indicates identified by MSMS fragments to the NIST spectral library.
