# Supplementary material for: Impact of Blood Collection Tubes and Sample Handling Time on Serum and Plasma Metabolome and Lipidome
Source: Metabolites. 2018 Dec 4;8(4):88. doi: 10.3390/metabo8040088 (PMC6316012; doi:10.3390/metabo8040088)
Supplement: Supplementary file 1 [file metabolites-08-00088-s001.zip › Supplemental Files-Proofreading/Supplemental Document S1 - Enrichment Results.pdf]

**Supplemental Document S1. (A) Taxonomy enrichment results for the statistically significant compounds across tubes**. Repeated measures ANOVA with Bonferroni FWER ≤ 0.05 and fold change ≥ 1.5 was used to identify differences between the P100, EDTA, and SST. Then the differences between each group were determined and the significant metabolites from each comparison that contained an identification or annotation, were exported to MBRole to determine whether certain chemical classes were more predominantly captured by specific tubes. Results were filtered for FDR ≤ 0.05 and sorted from most represented to least represented classes.

| Comparison | Chemical Taxonomy | Category | # in set | p-value | FDR |
|---|---|---|---|---|---|
| P100 vs. EDTA | Primary alcohol | HMDB | 17 | 2.63E-04 | 1.21E-02 |
| P100 vs. EDTA | Secondary carboxylic acid amide | HMDB | 11 | 2.04E-04 | 1.21E-02 |
| P100 vs. EDTA | Carboxamide group | HMDB | 11 | 7.48E-04 | 2.29E-02 |
| P100 vs. EDTA | Allyl alcohol | HMDB | 7 | 5.93E-04 | 2.18E-02 |
| P100 vs. EDTA | Amino Acids, Peptides, and Analogues | HMDB | 7 | 1.99E-03 | 4.58E-02 |
| P100 vs. EDTA | Fatty Alcohols | HMDB | 5 | 1.21E-04 | 1.11E-02 |
| P100 vs. EDTA | N-acyl-amine | HMDB | 5 | 1.35E-03 | 3.55E-02 |
| P100 vs. EDTA | Lysophosphatidylethanolamines | HMDB | 4 | 1.53E-06 | 2.82E-04 |
| P100 vs. EDTA | Sphingomyelins | Lipid Maps | 3 | 4.31E-05 | 2.11E-03 |
| EDTA vs. SST | Secondary alcohol | HMDB | 44 | 1.68E-04 | 4.75E-03 |
| EDTA vs. SST | Primary alcohol | HMDB | 32 | 3.61E-08 | 1.09E-05 |
| EDTA vs. SST | Glycerophospholipids | Lipid Maps | 24 | 4.36E-04 | 3.23E-02 |
| EDTA vs. SST | 1,2-Diol | HMDB | 21 | 3.54E-03 | 2.97E-02 |
| EDTA vs. SST | Cyclohexane | HMDB | 18 | 1.04E-03 | 1.31E-02 |
| EDTA vs. SST | Secondary carboxylic acid amide | HMDB | 17 | 1.42E-05 | 7.15E-04 |
| EDTA vs. SST | Carboxamide group | HMDB | 17 | 9.65E-05 | 3.24E-03 |
| EDTA vs. SST | Prenol Lipids | HMDB | 17 | 1.61E-03 | 1.58E-02 |
| EDTA vs. SST | Saccharide | HMDB | 16 | 3.11E-03 | 2.68E-02 |
| EDTA vs. SST | Bicyclohexane | HMDB | 11 | 1.31E-03 | 1.41E-02 |
| EDTA vs. SST | Allyl alcohol | HMDB | 10 | 1.73E-04 | 4.75E-03 |
| EDTA vs. SST | Sesterterpene | HMDB | 10 | 1.99E-04 | 5.01E-03 |
| EDTA vs. SST | Decaline | HMDB | 10 | 4.44E-03 | 3.12E-02 |
| EDTA vs. SST | Choline | HMDB | 9 | 2.13E-03 | 1.95E-02 |
| EDTA vs. SST | Quaternary ammonium salt | HMDB | 9 | 4.11E-03 | 3.12E-02 |
| EDTA vs. SST | Steroid | HMDB | 8 | 7.09E-05 | 2.68E-03 |
| EDTA vs. SST | Fatty Alcohols | HMDB | 7 | 1.80E-05 | 7.77E-04 |
| EDTA vs. SST | Polycyclic triterpene | HMDB | 7 | 1.28E-03 | 1.41E-02 |
| EDTA vs. SST | Triterpene | HMDB | 7 | 1.26E-03 | 1.41E-02 |
| EDTA vs. SST | Bile acid, alcohol, or derivative | HMDB | 6 | 1.35E-05 | 7.15E-04 |
| EDTA vs. SST | Hydroxy bile acid, alcohol, or derivative | HMDB | 6 | 1.19E-05 | 7.15E-04 |
| EDTA vs. SST | Sphingolipids | HMDB | 6 | 1.49E-03 | 1.55E-02 |
| EDTA vs. SST | 3-Hydroxy-steroid | HMDB | 6 | 1.67E-03 | 1.58E-02 |
| EDTA vs. SST | N-acyl-amine | HMDB | 6 | 2.66E-03 | 2.36E-02 |
| EDTA vs. SST | Alkyl glycoside | HMDB | 6 | 3.64E-03 | 2.97E-02 |
| EDTA vs. SST | Triterpene Glycosides | HMDB | 6 | 4.56E-03 | 3.13E-02 |
| EDTA vs. SST | Carboxylic acid salt | HMDB | 5 | 6.75E-04 | 1.07E-02 |
| EDTA vs. SST | 24-Hydroxy-steroid | HMDB | 4 | 4.25E-06 | 6.42E-04 |
| EDTA vs. SST | Lysophosphatidylethanolamines | HMDB | 4 | 1.24E-05 | 7.15E-04 |
| EDTA vs. SST | Hexose disaccharide | HMDB | 4 | 1.23E-03 | 1.41E-02 |
| EDTA vs. SST | Acyl Carnitines | HMDB | 3 | 2.33E-04 | 5.03E-03 |
| EDTA vs. SST | Carnitine | HMDB | 3 | 2.33E-04 | 5.03E-03 |
| EDTA vs. SST | Furostane-skeleton | HMDB | 3 | 2.88E-04 | 5.44E-03 |
| EDTA vs. SST | N-acyl Amines | HMDB | 3 | 5.23E-04 | 8.77E-03 |

| | | | | | | |
|---|---|---|---|---|---|---|
| EDTA vs. SST | Fatty Amides | HMDB | 3 | 7.96E-04 | 1.14E-02 |
| EDTA vs. SST | Unsaturated Fatty Acids | HMDB | 3 | 1.67E-03 | 1.58E-02 |
| EDTA vs. SST | Simple Glc series | Lipid Maps | 3 | 1.34E-03 | 3.92E-02 |
| P100 vs. SST | Secondary alcohol | HMDB | 37 | 1.51E-03 | 1.84E-02 |
| P100 vs. SST | Primary alcohol | HMDB | 27 | 1.04E-06 | 3.17E-04 |
| P100 vs. SST | Glycerophospholipids | Lipid Maps | 20 | 1.56E-03 | 2.03E-02 |
| P100 vs. SST | 1,2-Diol | HMDB | 19 | 3.84E-03 | 3.45E-02 |
| P100 vs. SST | Prenol Lipids | HMDB | 17 | 3.49E-04 | 8.42E-03 |
| P100 vs. SST | Cyclohexane | HMDB | 17 | 6.05E-04 | 1.04E-02 |
| P100 vs. SST | Cyclic alcohol | HMDB | 15 | 2.91E-04 | 8.42E-03 |
| P100 vs. SST | Secondary carboxylic acid amide | HMDB | 14 | 1.64E-04 | 7.15E-03 |
| P100 vs. SST | Carboxamide group | HMDB | 14 | 7.69E-04 | 1.12E-02 |
| P100 vs. SST | Saccharide | HMDB | 14 | 5.72E-03 | 4.09E-02 |
| P100 vs. SST | Bicyclohexane | HMDB | 11 | 4.36E-04 | 9.50E-03 |
| P100 vs. SST | Sesterterpene | HMDB | 10 | 6.68E-05 | 5.67E-03 |
| P100 vs. SST | Decaline | HMDB | 10 | 1.71E-03 | 2.01E-02 |
| P100 vs. SST | Drimane-skeleton | HMDB | 8 | 6.15E-04 | 1.04E-02 |
| P100 vs. SST | Allyl alcohol | HMDB | 8 | 1.41E-03 | 1.79E-02 |
| P100 vs. SST | Choline | HMDB | 8 | 3.45E-03 | 3.45E-02 |
| P100 vs. SST | Enone | HMDB | 8 | 4.61E-03 | 3.91E-02 |
| P100 vs. SST | Quaternary ammonium salt | HMDB | 8 | 6.22E-03 | 4.24E-02 |
| P100 vs. SST | Alkyl glycoside | HMDB | 7 | 3.20E-04 | 8.42E-03 |
| P100 vs. SST | 3-Hydroxy-steroid | HMDB | 6 | 8.54E-04 | 1.18E-02 |
| P100 vs. SST | Polycyclic triterpene | HMDB | 6 | 3.20E-03 | 3.37E-02 |
| P100 vs. SST | Triterpene | HMDB | 6 | 3.16E-03 | 3.37E-02 |
| P100 vs. SST | Hydroxy bile acid, alcohol, or derivative | HMDB | 5 | 8.40E-05 | 5.67E-03 |
| P100 vs. SST | Bile acid, alcohol, or derivative | HMDB | 5 | 9.30E-05 | 5.67E-03 |
| P100 vs. SST | Sphingolipids | HMDB | 5 | 4.60E-03 | 3.91E-02 |
| P100 vs. SST | Pimarane-skeleton | HMDB | 5 | 7.69E-03 | 4.55E-02 |
| P100 vs. SST | Steroid | HMDB | 5 | 7.20E-03 | 4.55E-02 |
| P100 vs. SST | Lysophosphatidylethanolamines | HMDB | 4 | 7.44E-06 | 1.13E-03 |
| P100 vs. SST | Fatty Alcohols | HMDB | 4 | 5.77E-03 | 4.09E-02 |
| P100 vs. SST | Fatty Acids and Conjugates | HMDB | 4 | 7.76E-03 | 4.55E-02 |
| P100 vs. SST | 24-hydroxy-steroid | HMDB | 3 | 1.19E-04 | 6.05E-03 |
| P100 vs. SST | N-acyl-amine | HMDB | 3 | 3.59E-04 | 8.42E-03 |
| P100 vs. SST | Fatty Amides | HMDB | 3 | 5.47E-04 | 1.04E-02 |
| P100 vs. SST | Sphingomyelins | Lipid Maps | 3 | 1.72E-04 | 1.12E-02 |
| P100 vs. SST | Unsaturated Fatty Acids | HMDB | 3 | 1.15E-03 | 1.52E-02 |
| P100 vs. SST | Simple Glc series | Lipid Maps | 3 | 8.23E-04 | 2.03E-02 |
| P100 vs. SST | 7-Hydroxy-steroid | HMDB | 3 | 6.25E-03 | 4.24E-02 |
| P100 vs. SST | Hexose disaccharide | HMDB | 3 | 7.98E-03 | 4.59E-02 |

**Supplemental Document S1. (B) Taxonomy enrichment results for all compounds across all tubes**. Taxonomy enrichment was performed all compounds with identifications and annotations detected in all tubes for all time points to obtain an unbiased and unfiltered list of all detected compounds. Metabolite accession IDs were imported into MBRole to determine the chemical classes that were more predominantly represented across the entire dataset. Results were filtered for FDR ≤ 0.05 and sorted from most represented to least represented classes.

| Chemical Taxonomy | Category | # in set | p-value | FDR |
|---|---|---|---|---|
| secondary alcohol | HMDB | 413 | 0.00E+00 | 0.00E+00 |
| Glycerophospholipids | Lipid Maps | 255 | 0.00E+00 | 0.00E+00 |
| primary alcohol | HMDB | 236 | 0.00E+00 | 0.00E+00 |
| 1,2-diol | HMDB | 213 | 0.00E+00 | 0.00E+00 |
| carboxylic acid | HMDB | 198 | 0.00E+00 | 0.00E+00 |
| cyclohexane | HMDB | 184 | 0.00E+00 | 0.00E+00 |
| dicarboxylic acid derivative | HMDB | 171 | 1.31E-03 | 8.83E-03 |
| saccharide | HMDB | 162 | 0.00E+00 | 0.00E+00 |
| Prenol Lipids | HMDB | 148 | 1.33E-15 | 1.07E-13 |
| oxane | HMDB | 141 | 6.60E-09 | 1.51E-07 |
| glycosyl compound | HMDB | 139 | 5.05E-12 | 1.62E-10 |
| acetal | HMDB | 133 | 1.15E-08 | 2.20E-07 |
| cyclic alcohol | HMDB | 126 | 2.66E-15 | 1.94E-13 |
| organic hypophosphite | HMDB | 125 | 2.08E-06 | 3.04E-05 |
| carboxamide_group | HMDB | 124 | 3.22E-15 | 2.15E-13 |
| o-glycosyl compound | HMDB | 116 | 1.10E-09 | 2.68E-08 |
| bicyclohexane | HMDB | 111 | 0.00E+00 | 0.00E+00 |
| secondary carboxylic acid amide | HMDB | 110 | 1.71E-14 | 1.06E-12 |
| decaline | HMDB | 97 | 0.00E+00 | 0.00E+00 |
| cyclohexene | HMDB | 90 | 1.11E-08 | 2.17E-07 |
| sesterterpene | HMDB | 82 | 0.00E+00 | 0.00E+00 |
| oxolane | HMDB | 75 | 3.55E-07 | 5.70E-06 |
| isoprene | HMDB | 73 | 2.15E-08 | 4.01E-07 |
| Glycerophosphocholines | Lipid Maps | 70 | 1.35E-13 | 5.60E-12 |
| allyl alcohol | HMDB | 67 | 4.71E-14 | 2.52E-12 |
| Amino Acids, Peptides, and Analogues | HMDB | 62 | 3.76E-08 | 6.86E-07 |
| quaternary ammonium salt | HMDB | 60 | 5.93E-07 | 9.16E-06 |
| drimane-skeleton | HMDB | 58 | 4.52E-12 | 1.51E-10 |
| Steroids and Steroid Derivatives | HMDB | 55 | 1.04E-12 | 3.98E-11 |
| tertiary alcohol | HMDB | 55 | 2.71E-05 | 3.25E-04 |
| triterpene | HMDB | 52 | 1.03E-13 | 4.87E-12 |
| polycyclic triterpene | HMDB | 52 | 1.15E-13 | 5.13E-12 |
| enone | HMDB | 52 | 4.03E-05 | 4.69E-04 |
| Diacylglycerols | HMDB | 51 | 1.23E-05 | 1.62E-04 |
| Glycerophosphates | Lipid Maps | 50 | 1.50E-11 | 4.15E-10 |
| choline | HMDB | 49 | 8.73E-05 | 9.33E-04 |
| Triterpene Glycosides | HMDB | 48 | 2.26E-12 | 8.25E-11 |
| hexose monosaccharide | HMDB | 46 | 1.61E-05 | 2.03E-04 |
| Glycerophosphoinositols | Lipid Maps | 44 | 1.03E-08 | 2.14E-07 |
| Diacylglycerophosphocholines | Lipid Maps | 43 | 9.91E-07 | 1.27E-05 |
| steroid | HMDB | 40 | 9.85E-10 | 2.47E-08 |
| alkyl glycoside | HMDB | 40 | 7.65E-09 | 1.66E-07 |
| 3-hydroxy-steroid | HMDB | 39 | 2.90E-10 | 7.51E-09 |
| Sphingolipids | HMDB | 37 | 1.86E-09 | 4.39E-08 |
| n-acyl-amine | HMDB | 36 | 1.17E-07 | 2.09E-06 |

| | | | | |
|---|---|---|---|---|
| pimarane-skeleton | HMDB | 36 | 1.42E-07 | 2.48E-06 |
| alpha-amino acid or derivative | HMDB | 36 | 5.32E-05 | 6.02E-04 |
| Phosphosphingolipids | Lipid Maps | 35 | 0.00E+00 | 0.00E+00 |
| Fatty Acids and Conjugates | HMDB | 35 | 3.14E-12 | 1.10E-10 |
| beta-hydroxy acid | HMDB | 35 | 1.46E-04 | 1.41E-03 |
| pyrimidine | HMDB | 35 | 2.18E-04 | 1.99E-03 |
| Amino Acids and Derivatives | HMDB | 34 | 9.56E-06 | 1.30E-04 |
| Glycerophosphoglycerols | Lipid Maps | 34 | 1.46E-04 | 9.70E-04 |
| triose monosaccharide | HMDB | 33 | 4.22E-14 | 2.42E-12 |
| carboxylic acid salt | HMDB | 33 | 8.00E-14 | 4.02E-12 |
| Diacylglycerophosphates | Lipid Maps | 33 | 4.27E-06 | 4.69E-05 |
| imidazole | HMDB | 33 | 1.33E-04 | 1.30E-03 |
| Glycerophosphoserines | Lipid Maps | 33 | 2.38E-04 | 1.46E-03 |
| seco-podocarpan-skeleton | HMDB | 30 | 2.67E-11 | 7.94E-10 |
| Diacylglycerophosphoinositols | Lipid Maps | 29 | 1.49E-04 | 9.70E-04 |
| Diacylglycerophosphoglycerols | Lipid Maps | 27 | 7.89E-04 | 4.68E-03 |
| monosaccharide phosphate | HMDB | 26 | 5.74E-06 | 8.09E-05 |
| n-acyl-alpha-amino-acid | HMDB | 26 | 9.75E-04 | 7.07E-03 |
| Diacylglycerophosphoserines | Lipid Maps | 26 | 1.61E-03 | 8.91E-03 |
| Peptides | HMDB | 26 | 3.24E-03 | 1.89E-02 |
| hydroxy bile acid, alcohol, or derivative | HMDB | 25 | 3.14E-11 | 9.01E-10 |
| Other Glycerophosphocholines | HMDB | 25 | 1.03E-08 | 2.07E-07 |
| pyrrole | HMDB | 25 | 8.07E-04 | 6.06E-03 |
| aminopyrimidine | HMDB | 25 | 9.77E-04 | 7.07E-03 |
| bile acid, alcohol, or derivative | HMDB | 24 | 2.84E-10 | 7.51E-09 |
| Fatty Alcohols | HMDB | 24 | 1.77E-06 | 2.63E-05 |
| n-glycosyl compound | HMDB | 24 | 1.62E-05 | 2.03E-04 |
| Alpha Amino Acids and Derivatives | HMDB | 24 | 1.96E-05 | 2.42E-04 |
| purine | HMDB | 24 | 8.03E-05 | 8.71E-04 |
| imidazopyrimidine | HMDB | 24 | 1.30E-04 | 1.29E-03 |
| ceramide | HMDB | 24 | 2.51E-04 | 2.21E-03 |
| fatty acyl glycoside | HMDB | 23 | 1.64E-03 | 1.05E-02 |
| Triterpenes | HMDB | 21 | 4.54E-05 | 5.21E-04 |
| pyrrolidine | HMDB | 21 | 8.83E-04 | 6.57E-03 |
| organic pyrophosphate | HMDB | 21 | 1.62E-03 | 1.04E-02 |
| sugar acid | HMDB | 21 | 7.83E-03 | 4.00E-02 |
| Ceramide phosphocholines (sphingomyelins) | Lipid Maps | 20 | 0.00E+00 | 0.00E+00 |
| 7-hydroxy-steroid | HMDB | 20 | 7.17E-09 | 1.60E-07 |
| cyclitol derivative | HMDB | 20 | 5.96E-03 | 3.28E-02 |
| inositol-phosphate | HMDB | 19 | 6.55E-04 | 5.11E-03 |
| pentacyclic triterpene | HMDB | 18 | 3.07E-04 | 2.62E-03 |
| 1-phosphoribosyl-imidazole | HMDB | 18 | 1.08E-03 | 7.55E-03 |
| pentose monosaccharide | HMDB | 18 | 3.47E-03 | 2.00E-02 |
| fatty alcohol | HMDB | 17 | 2.61E-05 | 3.18E-04 |
| diterpene | HMDB | 16 | 2.17E-07 | 3.68E-06 |
| farnesane-skeleton | HMDB | 16 | 5.14E-07 | 8.09E-06 |
| oleanane-skeleton | HMDB | 16 | 9.48E-04 | 6.98E-03 |
| Acyl Carnitines | HMDB | 15 | 2.99E-13 | 1.20E-11 |
| carnitine | HMDB | 15 | 2.99E-13 | 1.20E-11 |
| Unsaturated Fatty Acids | HMDB | 15 | 8.03E-09 | 1.70E-07 |
| organic sulfuric acid monoester | HMDB | 15 | 1.38E-05 | 1.79E-04 |
| hexose trisaccharide | HMDB | 15 | 1.76E-04 | 1.68E-03 |

| | | | | |
|---|---|---|---|---|
| Lysophosphatidylethanolamines | HMDB | 14 | 6.37E-11 | 1.76E-09 |
| Fatty amides | Lipid Maps | 14 | 2.31E-06 | 2.74E-05 |
| indole | HMDB | 14 | 2.69E-04 | 2.35E-03 |
| steroidal glycoside | HMDB | 14 | 2.14E-03 | 1.33E-02 |
| Anthocyanidins | Lipid Maps | 14 | 5.54E-03 | 2.42E-02 |
| lactam | HMDB | 14 | 6.44E-03 | 3.45E-02 |
| 1,2-aminoalcohol | HMDB | 14 | 8.87E-03 | 4.34E-02 |
| Monoacylglycerols | HMDB | 13 | 6.62E-12 | 2.04E-10 |
| Monoacylglycerophosphocholines | Lipid Maps | 13 | 1.28E-11 | 4.15E-10 |
| Ceramide phosphoethanolamines | Lipid Maps | 13 | 1.01E-09 | 2.40E-08 |
| tetracyclic triterpene | HMDB | 13 | 2.20E-07 | 3.68E-06 |
| sulfate-ester | HMDB | 13 | 3.02E-05 | 3.57E-04 |
| hexose disaccharide | HMDB | 13 | 6.49E-04 | 5.11E-03 |
| amphetamine or derivative | HMDB | 13 | 1.60E-03 | 1.04E-02 |
| primary carboxylic acid amide | HMDB | 13 | 3.16E-03 | 1.85E-02 |
| cinnamic acid or derivative | HMDB | 13 | 7.32E-03 | 3.77E-02 |
| hydroxyeicosapolyenoic acid | HMDB | 13 | 8.66E-03 | 4.34E-02 |
| Phosphatidylglycerols | HMDB | 12 | 1.04E-05 | 1.39E-04 |
| Phosphatidylinositols | HMDB | 12 | 6.88E-05 | 7.67E-04 |
| Diterpenes | HMDB | 12 | 2.76E-04 | 2.38E-03 |
| Eicosanoids | HMDB | 12 | 1.09E-03 | 7.55E-03 |
| 1-(1Z-alkenyl),2-acylglycerophosphates | Lipid Maps | 11 | 9.19E-08 | 1.39E-06 |
| N-acyl Amines | HMDB | 11 | 3.07E-07 | 5.03E-06 |
| Fatty Amides | HMDB | 11 | 1.36E-06 | 2.06E-05 |
| Ceramides | Lipid Maps | 11 | 2.96E-05 | 2.23E-04 |
| pinguisane-skeleton | HMDB | 11 | 7.32E-05 | 8.05E-04 |
| terpene glycoside | HMDB | 11 | 1.11E-03 | 7.55E-03 |
| Quinolines and Derivatives | HMDB | 11 | 2.09E-03 | 1.32E-02 |
| Acyl CoAs | HMDB | 11 | 8.46E-03 | 4.29E-02 |
| 24-keto-steroid | HMDB | 10 | 8.68E-09 | 1.79E-07 |
| Sesterterpenes | HMDB | 10 | 1.33E-03 | 8.83E-03 |
| C40 isoprenoids (tetraterpenes) | Lipid Maps | 10 | 2.60E-03 | 1.31E-02 |
| coenzyme_a | HMDB | 10 | 5.89E-03 | 3.26E-02 |
| enamine | HMDB | 10 | 9.38E-03 | 4.54E-02 |
| Hydroxy Fatty Acids | HMDB | 9 | 3.12E-06 | 4.47E-05 |
| Sphingoid bases | Lipid Maps | 9 | 4.67E-06 | 4.69E-05 |
| sulfonic acid | HMDB | 9 | 1.01E-04 | 1.04E-03 |
| 12-hydroxy-steroid | HMDB | 9 | 7.67E-04 | 5.81E-03 |
| N-acylsphingosines (ceramides) | Lipid Maps | 8 | 6.50E-05 | 4.69E-04 |
| dihydroxy bile acid, alcohol, or derivative | HMDB | 8 | 1.97E-04 | 1.84E-03 |
| Cholesteryl Esters | HMDB | 7 | 1.13E-04 | 1.13E-03 |
| monohydroxy bile acid, alcohol, or derivative | HMDB | 7 | 3.12E-04 | 2.64E-03 |
| Simple Glycosylceramides | HMDB | 7 | 5.57E-04 | 4.47E-03 |
| n-acylglycine | HMDB | 7 | 1.01E-03 | 7.24E-03 |
| Dicarboxylic Acids and Derivatives | HMDB | 7 | 2.95E-03 | 1.74E-02 |
| 1-alkyl,2-acylglycerophosphocholines | Lipid Maps | 7 | 7.15E-03 | 2.88E-02 |
| Monoacylglycerophosphoinositols | Lipid Maps | 6 | 5.30E-06 | 4.89E-05 |
| Ceramides | HMDB | 6 | 2.18E-04 | 1.99E-03 |
| trihydroxy bile acid, alcohol, or derivative | HMDB | 6 | 2.50E-04 | 2.21E-03 |
| 24-hydroxy-steroid | HMDB | 6 | 5.88E-04 | 4.67E-03 |
| 25-hydroxy-steroid | HMDB | 6 | 1.09E-03 | 7.55E-03 |
| Diterpene Glycosides | HMDB | 6 | 1.32E-03 | 8.83E-03 |

| | | | | |
|---|---|---|---|---|
| furostane-skeleton | HMDB | 6 | 1.45E-03 | 9.47E-03 |
| Dicarboxylic acids | Lipid Maps | 6 | 6.93E-03 | 2.88E-02 |
| Tetrapyrroles and Derivatives | HMDB | 6 | 7.26E-03 | 3.76E-02 |
| N-acyl amines | Lipid Maps | 6 | 1.43E-02 | 4.84E-02 |
| Diacylglycerophosphoglycerophosphodiradylglycerols | Lipid Maps | 5 | 1.17E-08 | 2.16E-07 |
| Oxidized glycerophosphocholines | Lipid Maps | 5 | 3.08E-08 | 5.11E-07 |
| Glycerophosphoglycerophosphoglycerols | Lipid Maps | 5 | 4.15E-07 | 5.74E-06 |
| Glycinated Bile Acids and Derivatives | HMDB | 5 | 7.39E-06 | 1.02E-04 |
| Oxidized glycerophospholipids | Lipid Maps | 5 | 2.92E-05 | 2.23E-04 |
| 23-keto-steroid | HMDB | 5 | 1.06E-04 | 1.08E-03 |
| Peptidomimetics | HMDB | 5 | 1.37E-03 | 9.02E-03 |
| Arthro and Ganglio series | HMDB | 5 | 6.26E-03 | 3.40E-02 |
| Steryl esters | Lipid Maps | 5 | 8.89E-03 | 3.43E-02 |
| 1-(1Z-alkenyl),2-acylglycerophosphoinositols | Lipid Maps | 5 | 1.06E-02 | 3.83E-02 |
| 1-alkyl,2-acylglycerophosphates | Lipid Maps | 5 | 1.06E-02 | 3.83E-02 |
| Fatty acyl carnitines | Lipid Maps | 4 | 4.80E-06 | 4.69E-05 |
| Primary amides | Lipid Maps | 4 | 1.82E-05 | 1.59E-04 |
| Thromboxanes | Lipid Maps | 4 | 2.60E-05 | 2.16E-04 |
| lanostane | HMDB | 4 | 8.83E-05 | 9.33E-04 |
| Aldehydes | HMDB | 4 | 2.31E-04 | 2.08E-03 |
| ergostane-skeleton | HMDB | 4 | 7.54E-04 | 5.77E-03 |
| macrolide | HMDB | 4 | 1.10E-03 | 7.55E-03 |
| Glycosyldiacylglycerols | Lipid Maps | 4 | 2.03E-03 | 1.05E-02 |
| sugar alcohol | HMDB | 4 | 2.10E-03 | 1.32E-02 |
| Glycosyldiradylglycerols | Lipid Maps | 4 | 3.49E-03 | 1.66E-02 |
| Straight chain fatty acids | Lipid Maps | 4 | 5.54E-03 | 2.42E-02 |
| alkanesulfonic acid derivative | HMDB | 4 | 4.57E-03 | 2.57E-02 |
| N-acyl ethanolamines (endocannabinoids) | Lipid Maps | 4 | 7.28E-03 | 2.88E-02 |
| organic transition metal moeity | HMDB | 4 | 5.70E-03 | 3.18E-02 |
| Sphingomyelins | HMDB | 4 | 8.49E-03 | 4.29E-02 |
| Straight Chain Fatty Acids | HMDB | 4 | 9.31E-03 | 4.53E-02 |
| Sphingoid base 1-phosphates | Lipid Maps | 3 | 1.52E-04 | 9.70E-04 |
| Thromboxanes | HMDB | 3 | 9.32E-05 | 9.72E-04 |
| Peptoid-Peptide Hydrids | HMDB | 3 | 1.84E-04 | 1.74E-03 |
| Glycosylglycerols | HMDB | 3 | 4.98E-04 | 4.04E-03 |
| Tetrahydroxy Bile Acids, Alcohols and Derivatives | HMDB | 3 | 7.35E-04 | 5.68E-03 |
| dipyrrin | HMDB | 3 | 1.03E-03 | 7.32E-03 |
| Taurinated Bile Acids and Derivatives | HMDB | 3 | 2.94E-03 | 1.74E-02 |
| Hydroquinolones | HMDB | 3 | 2.94E-03 | 1.74E-02 |
| Purine Ribonucleoside Triphosphates | HMDB | 3 | 2.94E-03 | 1.74E-02 |
| oxetane | HMDB | 3 | 3.62E-03 | 2.08E-02 |
| stigmastane-skeleton | HMDB | 3 | 4.38E-03 | 2.48E-02 |
| alpha-amino acid amide | HMDB | 3 | 6.19E-03 | 3.38E-02 |
| Glycoamino Acids and Derivatives | HMDB | 3 | 7.23E-03 | 3.76E-02 |
| Steroidal Glycosides | HMDB | 3 | 7.23E-03 | 3.76E-02 |
| Naphthyridines | HMDB | 3 | 7.23E-03 | 3.76E-02 |
| GalNAcb1-3Gala1-4Galb1-4Glc- (Globo series) | HMDB | 3 | 7.23E-03 | 3.76E-02 |
| benzimidazole | HMDB | 3 | 9.62E-03 | 4.63E-02 |
