# Supplementary material for: Impact of Blood Collection Tubes and Sample Handling Time on Serum and Plasma Metabolome and Lipidome
Source: Metabolites. 2018 Dec 4;8(4):88. doi: 10.3390/metabo8040088 (PMC6316012; doi:10.3390/metabo8040088)
Supplement: Supplementary file 1 [file metabolites-08-00088-s001.zip › Supplemental Files-Proofreading/Supplemental Document S2 - P100 and Tiger not EDTA.pdf]

Supplemental Document S2. Present in P100 and SST but absent in EDTA. Some of the common compounds to P100 and SST from Figure 1 C and D and that were database annotated are listed alphabetically.

Compound Name	Compound Info.	RT (mins)	Formula	CAS	KEGG	HMDB	Lipid Maps	Fraction
1-Hexadecanol	Fatty alcohol	3.588	C16 H34 O	36653-82-4	C00823	HMDB03424	--	Lipid
2-Methyl-(Z)-7-octadecene	Fatty acyl	1.768	C19 H38	--	--	--	LMFA11000339	Lipid
3-[(2,6-Dichlorobenzylidene)amino]-6H-dibenzo[b,d]pyran-6-one	Coumarin	1.712	C20 H11 Cl2 N O2	--	C14949	--	--	Lipid
8-Iodocatechin Tetramethyl Ether	Flavonoid	0.409	C19 H21 I O6	--	--	--	--	Lipid
Agavoside A	Sterol lipid / Phytochemical	2.321	C33 H52 O9	56857-65-9	C08885	HMDB34391	--	Lipid
Ajoene	Organosulfur compound found in garlic	0.401	C9 H14 O S3	--	C16757	--	--	Lipid
Alpha-CEHC	Vitamin E antioxidant	1.387	C16 H22 O4	4072-32-6	--	HMDB01518	--	Aqueous
erythro-7,9-Hexatriacontanediol	Fatty acyl found in fats, oils, herbs, spices	1.470	C36 H74 O2	193419-75-9	--	HMDB31284	--	Lipid
GlcCer(d18:0/14:0)	Sphingolipid	6.639	C38 H75 N O8	--	C01190		--	Lipid
Glycerol triundecanoate	Triacylglycerol	7.126	C36 H68 O6	13552-80-2	--	HMDB31089	--	Lipid
Megalomicin C2	Polyketide / Antibiotic	1.409	C49 H86 N2 O17	--	C11988	--	LMPK04000029	Lipid
Micrococcin	Macrocyclic peptide antibiotic	1.812	C48 H49 N13 O9 S6	67401-56-3	C12051	--	--	Aqueous
Neuromedin B (1-3)	Peptide found in human CNS and GI tract	0.861	C12 H22 N4 O5	--	--	HMDB13016	--	Lipid
N-Undecanoylglycine	Glycine derivative and minor fatty acid metabolite	10.019	C13 H25 N O3	--	--	HMDB13286	--	Aqueous
Octadecanol	Fatty alcohol	4.446	C18 H38 O	112-92-5	D01924	HMDB02350	--	Lipid
PE(38:5)	Glycerophospholipid	5.644	C43 H76 N O8 P	--	C00350	HMDB09261	--	Aqueous
PE(39:1)	Glycerophospholipid	9.266	C44 H86 N O8 P	--	C00350	HMDB09747	--	Aqueous
Phosalone	Insecticide and acaricide	0.354	C12 H15 Cl N O4 P S2	--	C11028	--	--	Lipid
Rizatriptan	Migraine drug	9.628	C15 H19 N5	145202-66-0	--	--	--	Aqueous
Theasapogenol A	Prenol lipid found in tea	1.441	C30 H50 O6	13844-22-9	--	HMDB34519	--	Aqueous
Torvoside G	Steroidal glycoside found in fruits	4.078	C34 H56 O9	184777-22-8	--	HMDB30337	--	Aqueous
