# Supplementary material for: Impact of Blood Collection Tubes and Sample Handling Time on Serum and Plasma Metabolome and Lipidome
Source: Metabolites. 2018 Dec 4;8(4):88. doi: 10.3390/metabo8040088 (PMC6316012; doi:10.3390/metabo8040088)

Supplemental Document S3. Compounds detected in blood collection tubes. Blood collection tubes containing phosphate buffered saline (PBS) instead of blood were extracted and analyzed to determine contribution from blood collection tubes in metabolomics samples. The data was filtered for compounds present in both replicates within each group after subtracting out PBS, LCMS-grade solvents and sample preparation blanks. The compounds detected in each 'blank' blood collection tube is shown below. Additional tubes were included in this analysis to determine whether other tubes reflected similar results. * indicates the tubes used to collect the blood samples in our current blood tube study. Numbers are designated by AQ+LIP or columns 3 and 4 in the table below.

Tube Type	Size	BD Part #	# Replicates	# Compounds detected	# Database annotated
EDTA (small)	2 mL	367841	2	95+162 = **257**	53+40 = **93**
**EDTA (large) ***	10 mL	366643	2	145+167 = **312**	76+42 = **118**
**P100 ***	8.5 mL	366448	2	214+235 = **449**	138+89 = **227**
SST (gold)	5 mL	367986	2	653+594 = **1247**	292+105 = **397**
**SST (red/grey) ***	10 mL	367985	2	725+590 = **1315**	326+102 = **428**

Overlap of blood collection tubes used in the study

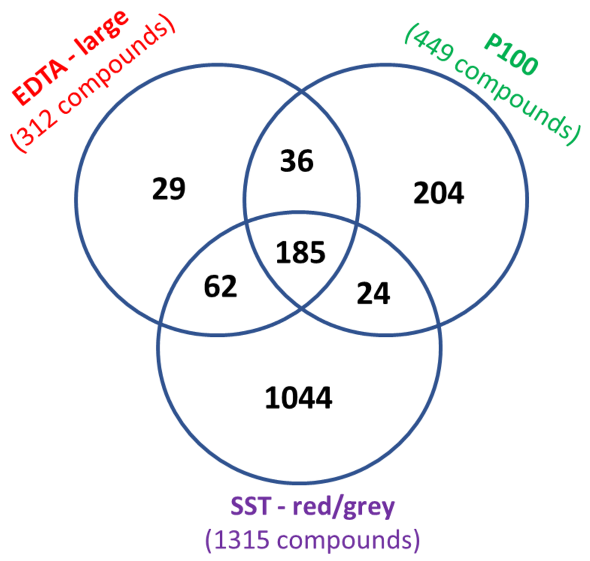

Comparison to other tubes of similar type

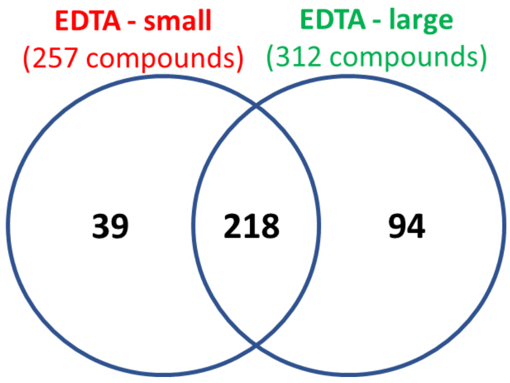

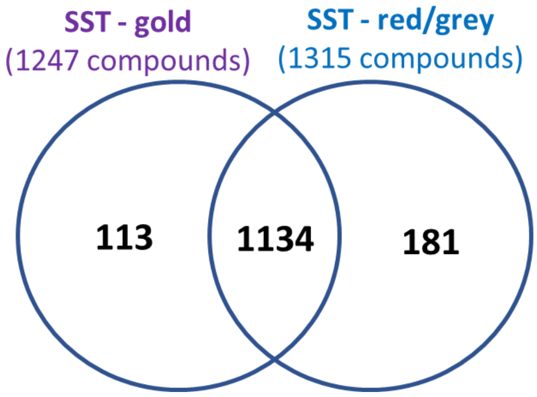

Supplement: Supplementary file 1 [file metabolites-08-00088-s001.zip › Supplemental Files-Proofreading/Supplemental Document S3 - Blank Vacutainers.pdf]
