# Supplementary material for: Impact of Blood Collection Tubes and Sample Handling Time on Serum and Plasma Metabolome and Lipidome
Source: Metabolites. 2018 Dec 4;8(4):88. doi: 10.3390/metabo8040088 (PMC6316012; doi:10.3390/metabo8040088)
Supplement: Supplementary file 1 [file metabolites-08-00088-s001.zip › Supplemental Files-Proofreading/Supplemental Document S4 - Unique to Tiger.pdf]

Supplemental Document S4. Unique to SST. The compounds that were only present in SST from Figure 1 C and D and that were database annotated are listed alphabetically.

Compound	Notes	RT (mins)	Formula	CAS	KEGG	HMDB	Lipid Maps
(24S)-7α,24-Dihydroxycholesterol	Bile acids	1.899	C27 H46 O3		C15518	HMDB60136	
(6S)-Hydroxyhyoscyamine	Alkaloids	0.628	C17 H23 N O4		C03325		
12-Tricosanol	Fatty alcohols	2.731	C23 H48 O	24897-74-3		HMDB34285	
17-Tetracosenal	Fatty aldehydes	9.308	C24 H46 O				LMFA06000105
20-O-Methyl-19-chloroproansamitocin	Antibiotic	0.698	C26 H34 Cl N O6		C20135		
2-Hydroxylauroylcarnitine	Acyl carnitines	4.417	C19 H37 N O5			HMDB13164	
3-trans-p-Coumaroylrotundic acid	Prenol lipids (triterpenoids)	1.623	C39 H54 O7	144624-03-3		HMDB40667	
5-Butyltetrahydro-2-oxo-3-furancarboxylic acid	Lactones	10.253	C9 H14 O4			HMDB30992	
5-Geranyloxy-7-methoxycoumarin	Prenol lipids (terpene lactones)	10.268	C20 H24 O4	7380-39-4		HMDB32954	
6-Carboxyhexanoyl-CoA	Biotin metabolism/benzoate degradation/microbial metabolism	1.194	C28 H46 N7 O19 P3 S		C01063		
6-O-Demethylnigerone	Naphthopyrans	0.679	C31 H24 O10	76045-68-6		HMDB33497	
Acebutolol	Beta blocking agents	0.647	C18 H28 N2 O4	37517-30-9	C06803		
Adenosylcobalamin	Tetrapyrroles	1.778	C72 H100 Co N18 O17 P	13870-90-1	C00194	HMDB02086	
Anatalline	Pyridines/Piperidines	1.284	C15 H17 N3		C12454		
Arachidonyl Trifluoromethyl Ketone	Ketones	9.380	C21 H31 F3 O	149301-79-1	C01397		
Arg Lys Tyr	Tripeptides	2.940	C21 H35 N7 O5				
Assamsaponin J	Terpene glycosides	1.218	C66 H96 O27	316157-19-4		HMDB36486	
Balhimycin	Glycopeptide antibiotic	1.812	C66 H73 Cl2 N9 O24		C15711		
Calpeptin	Amino acid amides	2.938	C20 H30 N2 O4	117591-20-5	C11256		
CL(70:5)	Cardiolipins	2.188	C79 H144 O17 P2		C05980	HMDB10240	
Crustecdysone	Dihydroxy bile acids	10.965	C27 H44 O7	5289-74-7	C02633	HMDB30180	
Cyclopassifloside III	Terpene glycosides	9.770	C43 H72 O16	292167-40-9		HMDB38390	
Cyclopassifloside X	Terpene glycosides	2.169	C37 H62 O12	301540-82-9		HMDB36365	
D-Fructose	Carbohydrates	10.571	C6 H12 O6	57-48-7	C10906	HMDB00660	
Ethiprole	Pesticide	1.225	C13 H9 Cl2 F3 N4 O S	181587-01-9	C18552		
GalNAcβ1-4GlcNAcβ-Sp	Carbohydrates (Oligosaccharides)	0.921	C18 H31 N5 O11				
Ganoderic acid K	Triterpenoids	2.123	C32 H46 O9	104700-95-0		HMDB35986	
Geranyl benzoate	Monoterpenoids	0.432	C17 H22 O2	94-48-4		HMDB33478	
Glu Cys Cys	Tripeptides	0.356	C11 H19 N3 O6 S2				
Hodgkinsine	Indole alkaloids	9.130	C33 H38 N6		C09211		
Icariin	Flavonoids	0.960	C33 H40 O15		C17555		

Inosine	Purine nucleosides	10.844	C10 H12 N4 O5	58-63-9	C00294	HMDB00195	
Isopentenyl adenosine	Nucleosides (phytohormone; drug)	2.306	C15 H21 N5 O4		C16427		
Leucomycin A1	Macrolides (Antibiotics)	2.199	C40 H67 N O14	16846-34-7	C14056		
Leupeptin	Peptides	10.964	C20 H38 N6 O4	55123-66-5	C01591		
Linoleyl carnitine	Acyl carnitines	2.185	C25 H45 N O4	36816-10-1		HMDB06469	
Lyciumoside IV	Terpene glycosides	0.713	C38 H64 O16	212773-31-4		HMDB33499	
LysoSM(d18:1)	Phosphosphingolipids	1.771	C23 H50 N2 O5 P	1670-26-4	C03640	HMDB06482	
Methyl (9Z)-8'-oxo-6,8'-diapo-6-carotenoate	Diterpenoids	2.717	C23 H28 O3	101034-51-9		HMDB31979	
MGDG(38:9)	Glycosyldiradylglycerols	9.796	C47 H72 O10		C03692		LMGL05010016
N-Desethylamiodarone	Metabolite of amiodarone	9.749	C23 H25 I2 N O3	96027-74-6			
Nicotinic acid adenine dinucleotide phosphate (NAADP)	Nucleotides	2.193	C21 H28 N6 O18 P3	5502-96-5	C13051	HMDB01179	
N-Methylanthraniloyl-CoA	Microbials	1.732	C29 H43 N8 O17 P3 S		C12092		
N-Oleoylethanolamine	Amines	1.894	C20 H39 N O2	111-58-0		HMDB02088	
Nonadeca-10(Z)-enoic acid	Fatty acids	5.848	C19 H36 O2	67228-95-9	C00174	HMDB13622	
PA(32:0)	Glycerophosphates	2.212	C35 H69 O8 P		C00416		LMGP10010889
PC(39:6)	Glycerophosphocholines	5.462	C47 H83 N O8 P				LMGP01011579
PC(21:1)	Glycerophosphocholines	8.906	C29 H57 N O8 P				LMGP01010830
PC(40:9)	Glycerophosphocholines	1.841	C48 H78 N O8 P		C00157	HMDB08732	
PC(P-42:1)	Glycerophosphocholines	2.193	C50 H98 N O7 P			HMDB11329	
PE(37:1)	Glycerophosphoethanolamines	3.319	C42 H82 N O8 P		C00350	HMDB09516	
PE(P-40:6)	Glycerophosphoethanolamines	4.459	C45 H78 N O7 P		C00350	HMDB09677	
PE(P-42:1)	Glycerophosphoethanolamines	3.380	C47 H92 N O7 P		C00350	HMDB09775	
PE(O-20:0)	Glycerophosphoethanolamines	9.682	C25 H54 N O6 P		C04476		LMGP02060005
PE(P-29:0)	Glycerophosphoethanolamines	2.196	C34 H68 N O7 P				LMGP02030008
Pelargonidin 3,5-di-(6-acetylglucoside)	Flavonoids	1.873	C31 H35 O17				LMPK12010045
Perindoprilat	ACE inhibitor	0.644	C17 H28 N2 O5			HMDB60574	
PG(34:0)	Glycerophosphoglycerols	2.932	C40 H79 O10 P			HMDB10572	
PG(30:4)	Glycerophosphoglycerols	9.721	C36 H63 O10 P				LMGP04010425
PGP(36:2)	Glycerophosphoglycerols	1.965	C42 H80 O13 P2			HMDB13549	
PGPC	Glycerophosphocholines	9.665	C29 H56 N O10 P	89947-79-5			
p-Hydroxyfosinoprilat	ACE inhibitor	0.446	C27 H42 N O7 P	113411-10-2			
p-Hydroxyphenylbutazone glucuronide	NSAID	1.284	C25 H28 N2 O9	57817-94-4			
PI(44:10)	Glycerophosphoinositols	2.201	C53 H83 O13 P				LMGP06010952
PI(O-42:6)	Glycerophosphoinositols	2.205	C51 H89 O12 P		C01194		LMGP06020001
p-Menthan-3-ol	Monoterpenoids	1.530	C10 H20 O	1490-04-6		HMDB35765	
Pro Asp Lys	Tripeptides	0.428	C15 H26 N4 O6				

PS(22:4)	Glycerophosphoserines	3.215	C28 H48 N O9 P				LMGP03050014
PS(O-38:0)	Glycerophosphoserines	3.298	C44 H88 N O9 P				LMGP03020051
Pyridoxal	Pyridines	9.329	C8 H9 N O3	66-72-8	C00250	HMDB01545	
Sapiol	Fatty alcohols	5.601	C34 H70 O	28484-70-0		HMDB33177	
Sarsaparilloside	Steroidal glycosides	2.196	C57 H96 O28	24333-07-1	C08911	HMDB30118	
Senegin II	Triterpenoids	2.165	C70 H104 O32	34366-31-9	C08978		
Stearoylethanolamide	Amines	1.445	C20 H41 N O2	111-57-9		HMDB13078	
Tetrahydrofolyl-[Glu](n)	Pterins	1.248	C24 H30 N8 O9		C03541	HMDB06826	
TG(56:1)	Triacylglycerols	1.757	C59 H112 O6			HMDB42367	
TG(61:14)	Triacylglycerols	1.847	C64 H96 O6				LMGL03012328
TG(o-58:6)	Triacylglycerols	2.197	C61 H108 O5		C00422	HMDB51102	
Torvoside E	Steroidal glycosides	1.952	C40 H66 O14			HMDB41530	
Tyr Lys Tyr	Tripeptides	0.698	C24 H32 N4 O6				
