# Supplementary material for: Impact of Blood Collection Tubes and Sample Handling Time on Serum and Plasma Metabolome and Lipidome
Source: Metabolites. 2018 Dec 4;8(4):88. doi: 10.3390/metabo8040088 (PMC6316012; doi:10.3390/metabo8040088)
Supplement: Supplementary file 1 [file metabolites-08-00088-s001.zip › Supplemental Files-Proofreading/Supplemental Document S5 - Quality Control Results.pdf]

Supplemental Document S5. Quality control analysis. Samples were pooled using the biological sample aliquots from P100, EDTA, and SST. These pooled human plasma samples were injected after every five samples and monitored for retention time and peak area reproducibility using spiked standards and endogenous compounds.

(A) CVs for spiked internal standards and randomly selected endogenous compounds.

Fraction	Compound Type	Compound Name	% CV RT	% CV Area
Hydrophobic	Internal Standard	Testosterone-d2	0.24	6.02
Hydrophobic	Internal Standard	Cis-10-nonadecenoic acid	0.34	3.62
Hydrophobic	Internal Standard	Ceramide (d18:1/17:0)	0.33	5.23
Hydrophobic	Endogenous	5-beta-Androsterone	0.24	6.18
Hydrophobic	Endogenous	LysoPE (16:0)	0.23	5.27
Hydrophobic	Endogenous	LysoPC (14:0)	0.21	4.82
Hydrophobic	Endogenous	PC (34:2)	0.24	4.69
Hydrophobic	Endogenous	PC (36:4)	0.22	4.12
Hydrophilic	Internal Standard	Creatinine-d3	0.81	5.97
Hydrophilic	Endogenous	Gamma-Butyrolactone	0.28	6.95
Hydrophilic	Endogenous	Creatinine	0.67	8.93
Hydrophilic	Endogenous	5-Aminopentanoic acid	0.23	8.89
Hydrophilic	Endogenous	Valine	0.23	8.66
Hydrophilic	Endogenous	Leucine	0.25	7.26
Hydrophilic	Endogenous	3-Pyridylacetic acid	0.44	8.21
Hydrophilic	Endogenous	1-Methylnicotinamide	0.82	9.77

(B) Variation of QCs compared to samples. PCA showing clustering of the QCs compared to the biological samples.

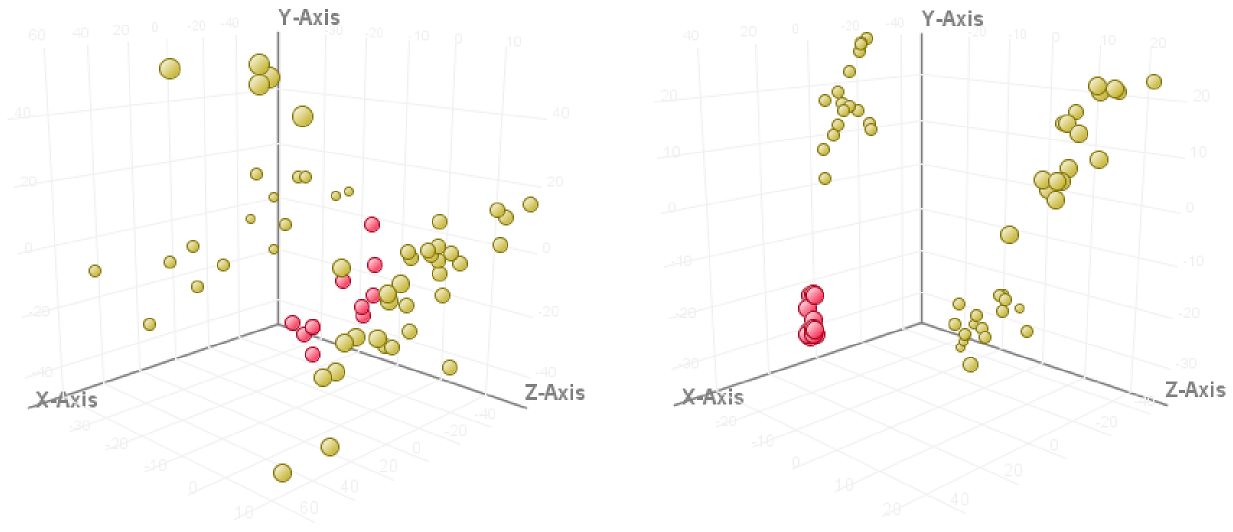

Hydrophilic

Hydrophobic
