# Supplementary figures and images for: Impact of Blood Collection Tubes and Sample Handling Time on Serum and Plasma Metabolome and Lipidome
Source: Metabolites. 2018 Dec 4;8(4):88. doi: 10.3390/metabo8040088 (PMC6316012; doi:10.3390/metabo8040088)

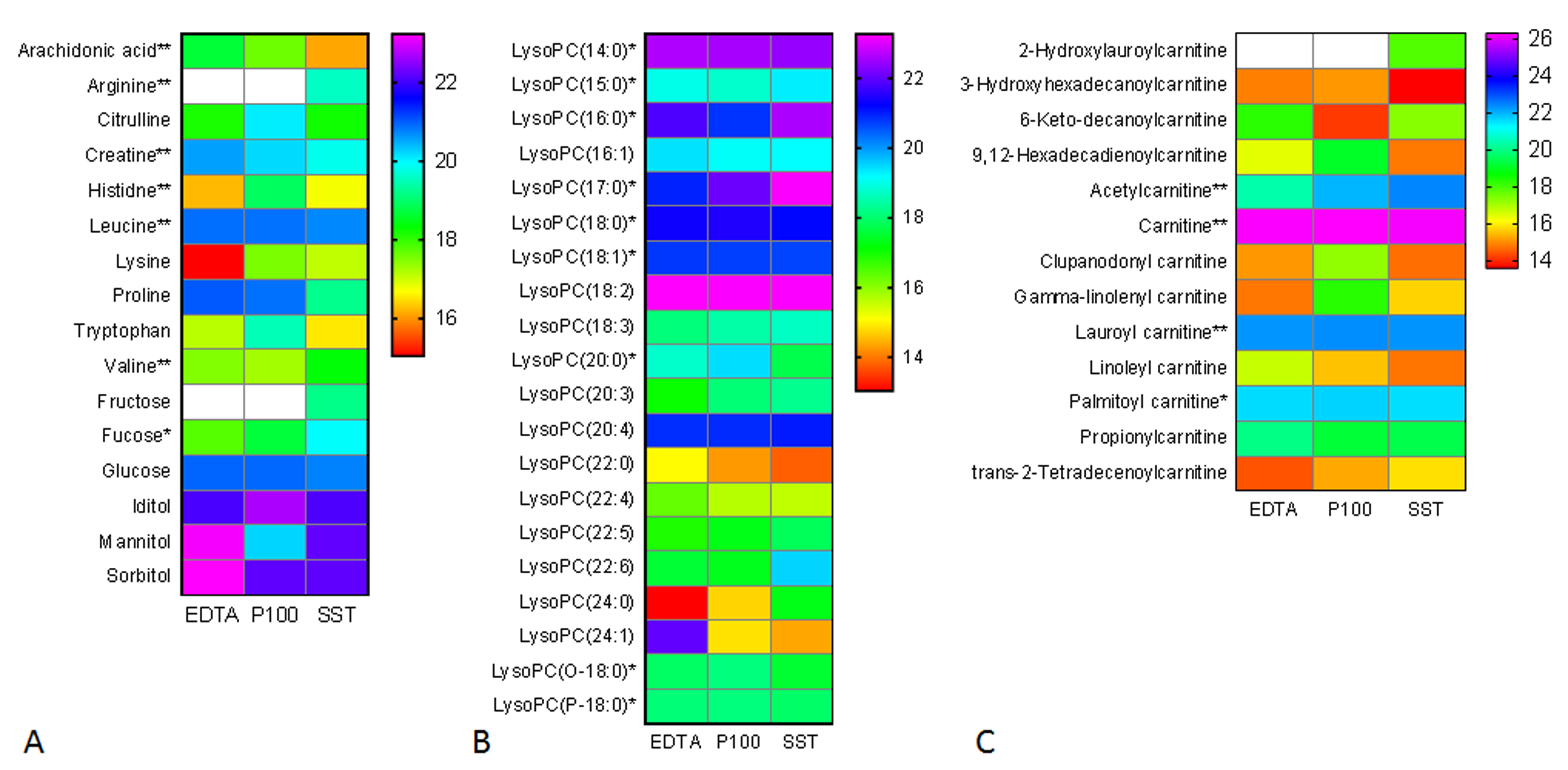

Supplement: Supplementary file 1 [file metabolites-08-00088-s001.zip › Supplemental Files-Proofreading/Supplemental Figure S1 - Abundance Trends.tif]

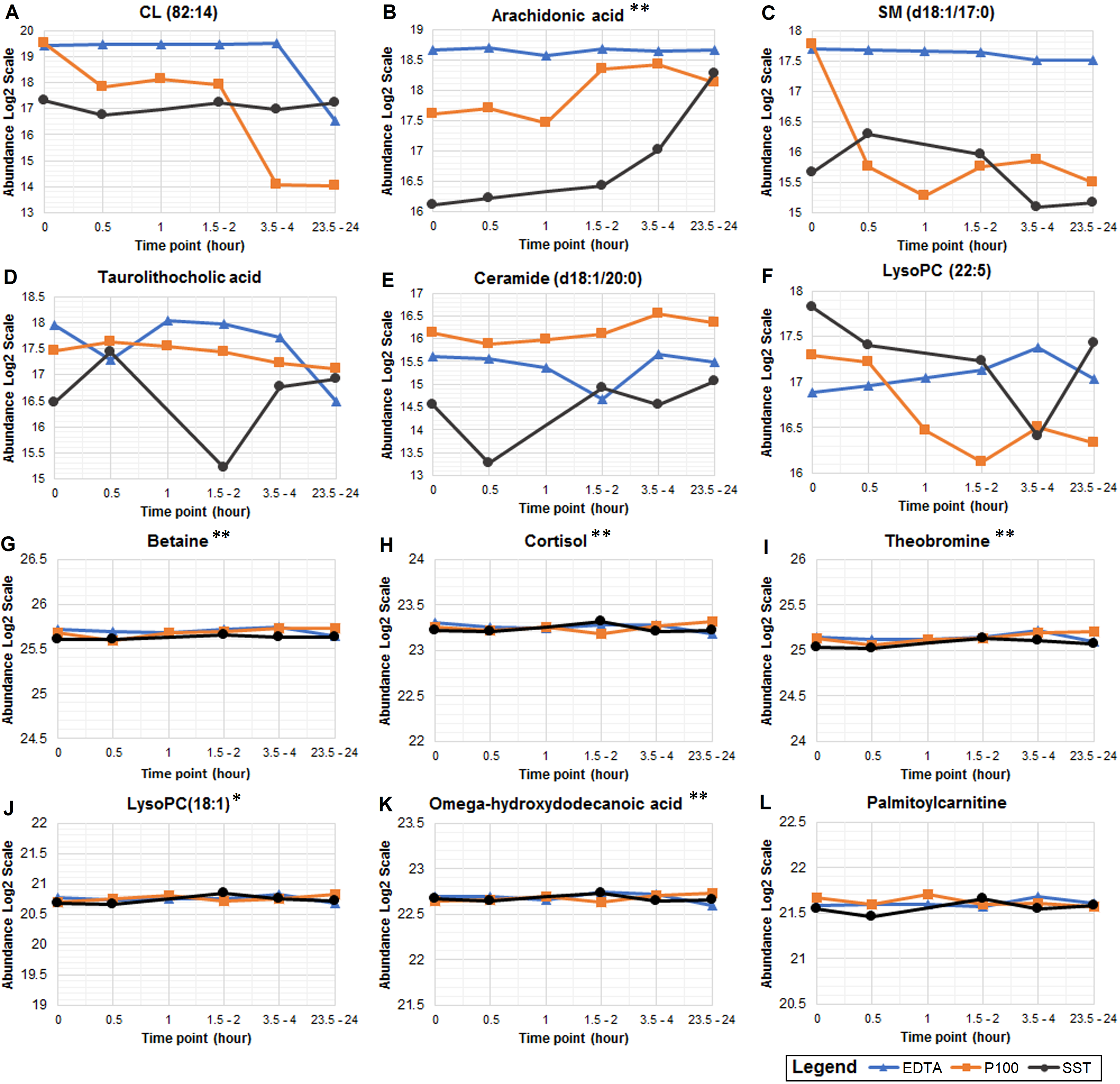

Supplement: Supplementary file 1 [file metabolites-08-00088-s001.zip › Supplemental Files-Proofreading/Supplemental Figure S2 - Time Trends per Tube_revisions.tif]
